# How Does Pyridoxamine Inhibit the Formation of Advanced Glycation End Products? The Role of Its Primary Antioxidant Activity

**DOI:** 10.3390/antiox8090344

**Published:** 2019-09-01

**Authors:** Rafael Ramis, Joaquín Ortega-Castro, Carmen Caballero, Rodrigo Casasnovas, Antonia Cerrillo, Bartolomé Vilanova, Miquel Adrover, Juan Frau

**Affiliations:** 1 Institut Universitari d’Investigació en Ciències de la Salut (IUNICS), Departament de Química, Universitat de les Illes Balears, 07122 Palma de Mallorca, Spain; 2 Institut d’Investigació Sanitària Illes Balears (IdISBa), 07120 Palma de Mallorca, Spain

**Keywords:** pyridoxamine, DFT, AGEs, inhibition, ROS

## Abstract

Pyridoxamine, one of the natural forms of vitamin B_6_, is known to be an effective inhibitor of the formation of advanced glycation end products (AGEs), which are closely related to various human diseases. Pyridoxamine forms stable complexes with metal ions that catalyze the oxidative reactions taking place in the advanced stages of the protein glycation cascade. It also reacts with reactive carbonyl compounds generated as byproducts of protein glycation, thereby preventing further protein damage. We applied Density Functional Theory to study the primary antioxidant activity of pyridoxamine towards three oxygen-centered radicals (•OOH, •OOCH_3_ and •OCH_3_) to find out whether this activity may also play a crucial role in the context of protein glycation inhibition. Our results show that, at physiological pH, pyridoxamine can trap the •OCH_3_ radical, in both aqueous and lipidic media, with rate constants in the diffusion limit (>1.0 × 10^8^ M${}^{-1}$ s${}^{-1}$). The quickest pathways involve the transfer of the hydrogen atoms from the protonated pyridine nitrogen, the protonated amino group or the phenolic group. Its reactivity towards •OOH and •OOCH_3_ is smaller, but pyridoxamine can still scavenge them with moderate rate constants in aqueous media. Since reactive oxygen species are also involved in the formation of AGEs, these results highlight that the antioxidant capacity of pyridoxamine is also relevant to explain its inhibitory role on the glycation process.

## 1. Introduction

Protein glycation involves a sequence of nonenzymatic reactions between proteins and sugars. The pathological complications of diabetes (kidney diseases [1], retinopathies [2], vascular diseases [3] and neurodegenerative diseases [4]) are directly related to the effects of these reactions on the structure and function of the affected proteins. The general mechanism of protein glycation starts with the addition of a nucleophilic group of a protein to the carbonyl group of glucose to form a Schiff base, which can subsequently yield an α-ketoamine known as an Amadori compound. Schiff bases and Amadori compounds can slowly and irreversibly evolve towards the so-called advanced glycation end products (AGEs) through a heterogeneous set of reactions [4,5,6].

AGEs can also be generated by several side reactions that occur in parallel to the general mechanism. These generally consist of metal-catalyzed oxidations of Schiff bases or Amadori compounds that yield reactive oxygen species (ROS) and reactive carbonyl species (RCS). Since ROS and RCS are much more reactive than glucose, their ability to damage proteins is much higher, as they enhance the number and type of AGEs that are formed [6,7,8,9].

Given the pathological implications of protein glycation, the design of molecules with the ability to attenuate its effects is a matter of utmost concern. The three main mechanisms by which these molecules, known as protein glycation inhibitors, prevent the formation of AGEs are: (i) the complexation of metal ions that catalyze the secondary reactions of protein glycation; (ii) the scavenging of RCS; and (iii) ROS generated as byproducts of those secondary reactions. In 1999, it was discovered that pyridoxamine (PM) had the ability of inhibiting the oxidation of Amadori compounds to form AGEs [10]. PM is one of the three natural forms of vitamin B_6_, together with pyridoxal (PL) and pyridoxine (PN). By that time, it was known that PM was very reactive towards carbonyl groups, since it acted as a coenzyme in transamination reactions between carbonyl compounds and amino groups [11], and this led to consider that it could be an effective AGE inhibitor. Thus far, several studies in both animal and human models have shown its therapeutic effects on diabetic kidney disease [12,13], as well as on retinopathies [14] and vascular diseases [15].

Concerning its mechanism of action, it has been suggested that PM could be effective as a metal chelating agent, a RCS scavenger and/or a ROS scavenger. It can form stable complexes with Cu^2+^ and Fe^3+^ [16,17] and stable adducts with several dicarbonyl compounds [18,19,20,21], as well as inhibit the production of the hydroxyl radical from the Fenton reaction or directly react with it [22,23,24]. Some studies also suggest that it can eliminate the superoxide radical anion from the medium [25], while others dispute this conclusion [24].

There exist several experimental assays to evaluate the antioxidant activity of molecules [26,27], but they neither distinguish between oxidant species nor allow the determination of the preferred mechanisms and reaction sites. For these reasons, theoretical methods such as DFT are particularly well suited to assess antioxidant activities. Some DFT studies on the reactivity of PN, another form of vitamin B_6_, towards hydroxyl, hydroperoxyl and superoxide were carried out by Matxain and coworkers [28,29]. Galano et al. also studied the antioxidant capacity of many different organic molecules by using this methodology [30,31,32,33,34].

Here, we aimed to study the ability of PM to scavenge ROS and to determine its preferred mechanisms and reaction sites by applying DFT. This would allow us to complete our previous studies on the formation of complexes between PM and Cu^2+^ or Fe^3+^ [35,36,37] and on its reactivity towards RCS [16,18,38,39] in the context of AGE inhibition. Specifically, thermodynamic and kinetic data were computed for all reaction paths between PM and the hydroxyl (•OOH), methylperoxyl (•OOCH_3_) and methoxyl (•OCH_3_) radicals, as well as their branching ratios (that is, the contribution of each path to the overall reactivity of PM). These radicals were chosen because using a highly reactive one such as hydroxyl could lead to the conclusion that all reaction paths are equally likely [40,41]. Three different mechanisms were studied: hydrogen-atom transfer, radical-adduct formation and single-electron transfer.

## 2. Materials and Methods

The quantum mechanics-based test for overall free radical scavenging activity (QM-ORSA) methodology, as explained by Galano et al. [42], was applied to compute the rate constants of the reactions between PM and the •OOH, •OOCH_3_ and •OCH_3_ radicals. Briefly, the geometries of PM and each radical, as well as the reaction products and transition states (TSs), were optimized at the M05-2X/6-311+G(d,p) level of theory. The unrestricted formalism was applied to open-shell systems, and the ultrafine integration grid was used in all the calculations. The M05-2X functional was designed for thermochemistry, thermochemical kinetics and noncovalent interactions [43], and successfully used for kinetic calculations and reaction energies in antioxidant-free radical systems [44,45,46]. After the geometry optimizations, frequencies were calculated to obtain Gibbs free energies for each species and to confirm the nature of the stationary points. Minima had no imaginary frequencies, while TSs had exactly one. The values of these imaginary frequencies are given in Tables S1 and S2. For each TS, the atom displacement associated to the imaginary frequency was coincident with the motion along the expected reaction coordinate.

**Figure 1:**
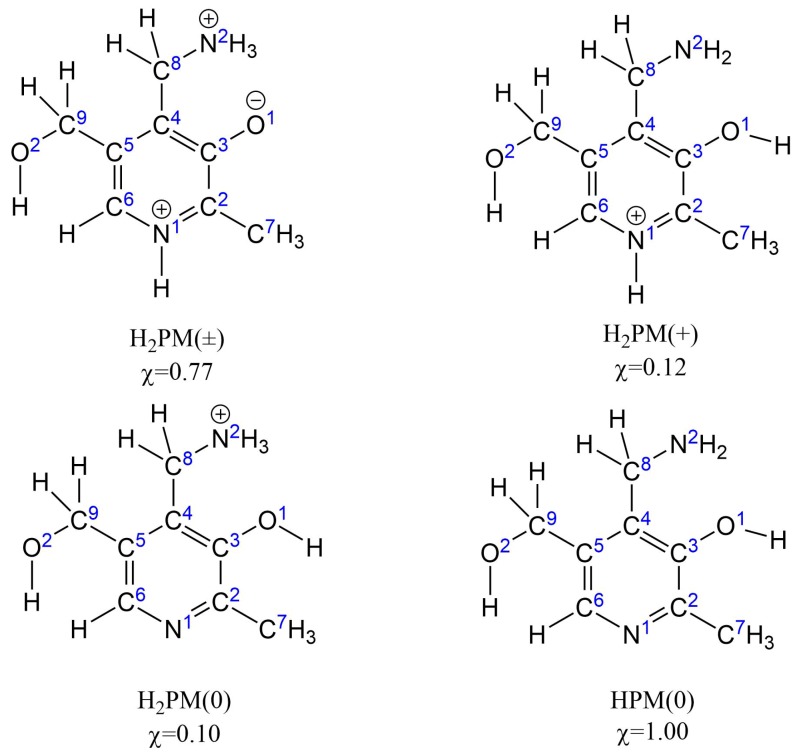
Atom numbering for the four pyridoxamine (PM) tautomers considered for calculations in water (H_2_PM(±), H_2_PM(+) and H_2_PM(0)) and pentyl ethanoate (HPM(0)). Mole fractions χ at physiological pH (7.4), calculated from the pK_a_ values collected by Casasnovas et al. [37], are also indicated.

Solvent effects were included in all geometry optimizations by using the universal SMD implicit solvation model [47]. Two different solvents, water and pentyl ethanoate, were used to mimic an aqueous and a lipidic microenvironment, respectively. The cationic form of PM, which is the predominant one at physiological pH (7.4) [48], was modeled in water. This form has three different tautomers with a significant mole fraction at pH 7.4 [37] (Figure 1) and all three were used in the calculations. In pentyl ethanoate, only the completely neutral form was considered. Three reaction mechanisms were studied: (i) the transfer of each hydrogen atom from PM to the radical (hydrogen-atom transfer, HAT); (ii) the formation of an adduct between PM and the radical at each aromatic atom (radical-adduct formation, RAF); and (iii) and the transfer of an electron (single-electron transfer, SET) from PM to the radical.

For each reaction, its standard reaction Gibbs free energy $\Delta G^{0}$ at 298.15 K was calculated as the difference between the Gibbs free energies of products and reactants, and only the reactions with a $\Delta G^{0}$ less than 1 kcal/mol were considered for the kinetic study. In these cases, activation Gibbs free energies $\Delta G^{\neq }$ at 298.15 K were calculated as the differences between the Gibbs free energies of TSs and reactants. These $\Delta G^{\neq }$ values are shown in Tables S3 and S4. All $\Delta G^{0}$ and $\Delta G^{\neq }$ values were referred to the standard state of 1 M. Solvent cage effects were considered by applying the corrections suggested by Okuno [49], based on the free volume theory [50].

Rate constants *k* were calculated by applying conventional transition state theory (TST) by using Equation (1):

(1) $$k=\kappa \sigma \frac{k_{B}T}{h}e^{-\frac{\Delta G^{\neq }}{RT}},$$

where $k_{B}$ is the Boltzmann constant; *h* is the Planck constant; *R* is the ideal gas constant; *T* is the absolute temperature; κ is the tunneling correction; and σ is the reaction path degeneracy, which takes into account the existence of different but equivalent reaction paths. Tunneling corrections κ were applied when computing the rate constants for the HAT reactions by considering an Eckart barrier [51], as implemented in the program by Brown et al. [52]. Tables S1 and S2 display the values of κ for all HAT reactions. For RAF reactions, no tunneling corrections were applied (κ = 1).

When rate constants *k* were above the diffusion limit (*k* > 1.0 × 10^8^ M${}^{-1}$ s${}^{-1}$), the Kimball–Collins theory [53] was used to correct them by applying Equation (2):

(2) $$k_{app}=\frac{k_{D}k}{k_{D}+k},$$

where $k_{D}$ is the steady-state Smoluchowski rate constant for a diffusion-controlled, bimolecular, irreversible reaction, which is obtained from Equation (3) [54]:

(3) $$k_{D}=4\pi RD_{AB}N_{A},$$

where *R* is the reaction distance; $N_{A}$ is the Avogadro number; and $D_{AB}$ is the mutual diffusion coefficient for the reactants A and B, calculated as the sum of their diffusion coefficients $D_{A}$ and $D_{B}$, which are calculated with the Stokes–Einstein approach, given by Equation (4) [55,56]:

(4) $$D_{i}=\frac{k_{B}T}{6\pi \eta a_{i}},$$

where $k_{B}$ is the Boltzmann constant; *T* is the absolute temperature; η is the solvent viscosity (8.91 × 10^−4^ Pa·s for water and 8.62 × 10^−4^ Pa·s for pentyl ethanoate); and $a_{i}$ is the radius of solute *i*, assuming it is spherical.

Overall rate constants for each combination of radical, solvent, mechanism and reaction site were calculated as the sums of the rate constants for each tautomer, weighted by their mole fractions $\chi_{i}$ at physiological pH (7.4), as shown in Equation (5):

(5) $$k_{overall}=\sum_{i=1}^{n}\chi_{i}k_{i},$$

where $k_{i}$ is the rate constant for the *i*th tautomer and *n* is the number of tautomers (3 for water and 1 for pentyl ethanoate). Total rate constants $k_{tot}$ were computed for each combination of radical and solvent as the sum of the overall rate constants for all mechanisms and reaction sites, as shown in Equation (6):

(6) $$k_{tot}=\sum_{}^{}k_{overall},$$

Branching ratios Γ were also calculated for each combination of radical, solvent, mechanism and reaction site by using Equation (7):

(7) $$\Gamma =\frac{100\cdot k_{overall}}{k_{tot}}.$$

All geometry optimizations and frequency calculations were performed with the Gaussian 09 package, revision D.01 [57].

## 3. Results

The thermodynamics and kinetics of the different reaction pathways between PM and the •OOH, •OOCH_3_ and •OCH_3_ radicals were determined and analyzed in this study, both in a polar solvent (water) and in a nonpolar solvent (pentyl ethanoate). Three mechanisms were considered:

- Hydrogen-atom transfer (HAT): PM + R•→ PM(-H)• + RH, considering each possible PM hydrogen atom.
- Radical-adduct formation (RAF): PM + R•→ [PM-R]•, at each aromatic PM atom.
- Single-electron transfer (SET): PM + R•→ PM^+^• + R${}^{-}$.

In water, the H_2_PM(±), H_2_PM(+) and H_2_PM(0) tautomers of the monocationic form of PM were considered, whereas only the neutral form HPM(0) was modeled in pentyl ethanoate (Figure 1).

### 3.1. Thermodynamic Study

Thermodynamically, •OCH_3_ is by far the most reactive of the three studied radicals in both solvents, and •OOH is slightly more reactive than •OOCH_3_ (Table 1). The standard reaction Gibbs free energies ($\Delta G^{0}$) for all HAT reactions in water involving •OCH_3_ are 18.5 kcal/mol lower than those with •OOH at the same position, and 19.1 kcal/mol lower than those with •OOCH_3_. $\Delta G^{0}$ values for RAF reactions in water involving •OCH_3_ are between 13.8 and 16.9 kcal/mol lower than the corresponding reactions involving •OOH and between 15.7 and 19.7 kcal/mol lower than those with •OOCH_3_. For SET reactions, the $\Delta G^{0}$ values for •OCH_3_ are also lower than those for the other two radicals, although the differences are smaller: 5.5 kcal/mol in the case of •OOH and 7.3 kcal/mol in the case of •OOCH_3_.

In pentyl ethanoate, the trends are the same (Table 2). $\Delta G^{0}$ values for HAT reactions with •OCH_3_ are 18.3 kcal/mol lower than those involving •OOH and 19.8 kcal/mol lower than those with •OOCH_3_. For RAF reactions, the differences are between 12.9 and 15.6 kcal/mol for •OOH and between 16.8 and 19.3 kcal/mol for •OOCH_3_. The $\Delta G^{0}$ for the SET reaction involving •OCH_3_ in pentyl ethanoate is 8.1 kcal/mol lower than that for •OOH and 9.4 kcal/mol lower than that for •OOCH_3_.

The most exergonic reaction pathway for the scavenging of •OCH_3_ in water is the abstraction of the C8 hydrogen atom of the H_2_PM(+) tautomer ($\Delta G^{0}$ = −31.2 kcal/mol). In general, the HAT reactions at the C7, C8 and C9 of all three tautomers in water are highly exergonic pathways for the scavenging of this radical. In addition, hydrogen abstractions from the N1 and N2 of H_2_PM(±) and from the O1 of H_2_PM(0), as well as the RAF reactions at the C2, C4 and C6 of H_2_PM(±), are thermodynamically favored pathways for the scavenging of •OCH_3_. There are other pathways that could also be thermodynamically possible, although with smaller negative (or even slightly positive) $\Delta G^{0}$ values: the HAT reactions at N2, O1 and O2 and the radical additions at C2, C4 and C6 of H_2_PM(+); and the HAT reaction at O2 and the radical additions at C2, C3, C4 and C6 of H_2_PM(0). In pentyl ethanoate, the trends do not vary much. The thermodynamically most favored mechanism to scavenge •OCH_3_ is the HAT reaction at C8 ($\Delta G^{0}$ = −18.5 kcal/mol), followed by the HAT reactions at C9, C7 and O1. The RAF reactions at C2, C3, C4 and C6, and the HAT reaction at N2 could be feasible as well, since their $\Delta G^{0}$ values are either slightly negative or slightly positive. All other pathways, such as the hydrogen abstractions from the sp^2^ carbon (C6) and especially the SET reactions, are clearly endergonic.

The most negative $\Delta G^{0}$ values for the scavenging of both •OOH and •OOCH_3_ in water also correspond to the abstraction of a hydrogen atom from the C8 of H_2_PM(+) (i.e., −12.7 and −12.1 kcal/mol, respectively). In pentyl ethanoate, the most favorable pathways for these two radicals are the same as in water, although with $\Delta G^{0}$ values close to 0 (−0.2 and 1.2 kcal/mol, respectively). All the other pathways either have $\Delta G^{0}$ values around 0 (i.e., hydrogen abstractions from the C7, C8, C9 and N1 atoms and radical additions to the C2, C4 and C6 atoms of H_2_PM(±); hydrogen abstraction from the C9 of H_2_PM(+) and, in the case of •OOCH_3_, from the O1 of H_2_PM(0)), or are clearly endergonic (e.g., hydrogen abstractions from the aromatic C6 atom and SET reactions).

In most cases, the radical adducts at N1 are not stable (their optimization results in their dissociation). In the few cases in which they do not dissociate, their formation is largely endergonic ($\Delta G^{0}$ values between 27.6 and 42.7 kcal/mol).

All pathways characterized as clearly endergonic ($\Delta G^{0}$ > 1 kcal/mol) were discarded as possible mechanisms to explain the ability of PM to scavenge ROS because, even if they took place at a significant rate, they would be reversible. Nevertheless, they would represent possible pathways if their products further reacted, in subsequent steps, with small activation barriers and high exergonicity.

### 3.2. Kinetic Study

**Figure 2:**
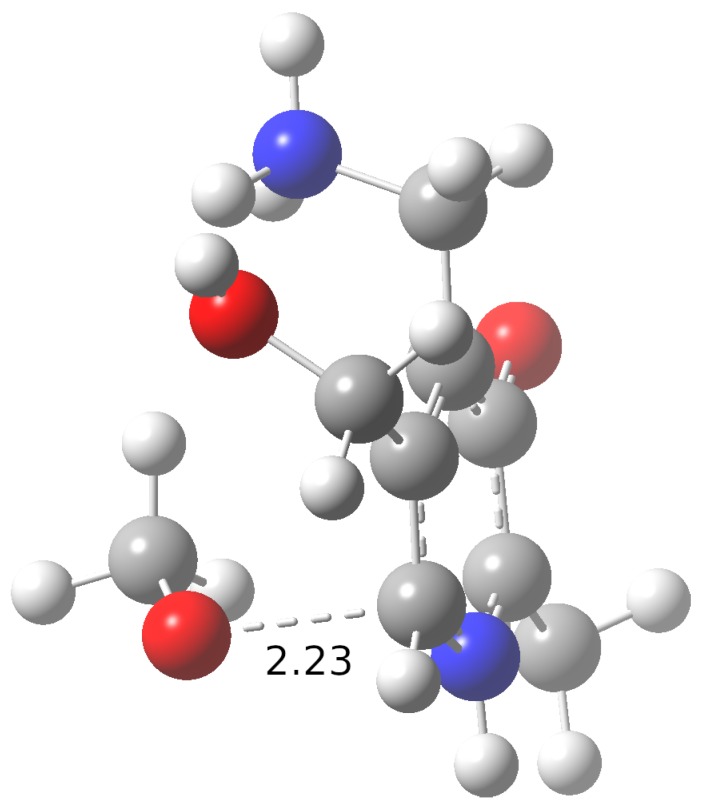
Transition state for the RAF reaction between the H_2_PM(±) tautomer of pyridoxamine and the •OCH_3_ radical at C6, which is the fastest reaction for which a transition state was located. The distance between the oxygen atom of •OCH_3_ and the C6 atom of H_2_PM(±) is given in Å.

Those reaction mechanisms with $\Delta G^{0}$ values below 1 kcal/mol were further analyzed from a kinetic point of view. The •OCH_3_ radical is the one exhibiting the highest rate constants. The H_2_PM(±) tautomer, which is predominant at physiological pH, is also the most reactive. It seems that •OCH_3_ may react with rate constants higher than the diffusion limit (>1.0 × 10^8^ M${}^{-1}$ s${}^{-1}$) by several mechanisms (Table 3 and Table 4): (i) via a HAT reaction at the N1 and N2 sites of the H_2_PM(±) tautomer and at the phenolic oxygen (O1) of the H_2_PM(+), H_2_PM(0) and HPM(0) tautomers; or (ii) through a RAF reaction at the C6 site of the H_2_PM(±) tautomer. For the mentioned HAT reactions involving the •OCH_3_ radical, no TS could be located at the level of theory used. However, a relaxed scan of the X-H distances (where X is N1, N2 or O1), leading from reactants to products, revealed a monotonic decrease of the energy (with no maximum along the way), suggesting that these reactions are strictly diffusion-controlled (i.e., every encounter between the reactants would yield the products). In contrast, we could find the TS corresponding to the diffusion-controlled •OCH_3_ RAF reaction at the C6 atom of H_2_PM(±) (Figure 2). The structures and the Cartesian coordinates of all TSs found in this study are given in the Supplementary Materials. Other pathways with high rate constants for the scavenging of •OCH_3_ are the formation of a radical adduct at the C2 and C4 positions of H_2_PM(±) (in the order of 10^7^ and 10^6^ M${}^{-1}$ s${}^{-1}$, respectively). Moderate rate constants are found for •OCH_3_ HAT reactions at the aliphatic carbon atoms (C7, C8 and C9) of all four tautomers (in the order of 10^3^–10^6^ M${}^{-1}$ s${}^{-1}$).

The other two radicals are not particularly reactive at any position, with only moderate rate constants for the RAF reactions at the C2 and C6 atoms of H_2_PM(±) (in the order of 10^4^ and 10^3^ M${}^{-1}$ s${}^{-1}$, respectively, for •OOH; and in the order of 10^4^ and 10^2^ M${}^{-1}$ s${}^{-1}$, respectively, for •OOCH_3_) and at its C4 atom (in the order of 10^2^ M${}^{-1}$ s${}^{-1}$ for •OOH). The rate constants at the remaining thermochemically viable sites are negligible (<10^2^ M${}^{-1}$ s${}^{-1}$). For the HAT reactions at the N1 atom of H_2_PM(±) involving •OOH and •OOCH_3_, and for the HAT reaction at the O1 atom of H_2_PM(0) involving •OOCH_3_, again no TSs could located at the level of theory used. However, as opposed to the case of •OCH_3_, a relaxed scan of the X-H distances (where X is N1 or O1) resulted in a monotonic increase of the energy (with no maximum along the way) when moving from reactants to products, suggesting that, in these cases, the inverse HAT reactions (i.e., the transfer of hydrogen from the radicals to PM) are the ones that are strictly diffusion-controlled. Therefore, these reactions were also discarded as viable pathways for the scavenging of these radicals.

PM would be able to scavenge the •OCH_3_ radical in an aqueous environment with a total rate constant ($k_{tot}$) of 5.3 × 10^9^ M${}^{-1}$ s${}^{-1}$ (Table 4), with HAT reactions at both nitrogen atoms N1 and N2 and at the phenolic oxygen O1 as the preferred reaction pathways (branching ratios of 42.0%, 41.2% and 12.4%, respectively). In a lipidic medium, the scavenging of •OCH_3_ would also be diffusion-controlled, with a $k_{tot}$ value of 3.0 × 10^9^ M${}^{-1}$ s${}^{-1}$, being the HAT reaction at the phenolic oxygen the unique non-negligible pathway. The other two radicals would be moderately scavenged in an aqueous environment, with $k_{tot}$ values of 3.2 × 10^4^ and 8.3 × 10^3^ M${}^{-1}$ s${}^{-1}$ for •OOH and •OOCH_3_, respectively. In the case of •OOH, the scavenging would take place mainly through the formation of radical adducts at the C2 and C6 atoms (branching ratios of 75.6% and 24.0%, respectively), and in the case of •OOCH_3_, the single preferred pathway would be the formation of a radical adduct at C2 (with a branching ratio of 98.0%). In a lipidic environment, however, PM would not efficiently scavenge •OOH or •OOCH_3_ ($k_{tot}$ values below 10^2^ M${}^{-1}$ s${}^{-1}$; for •OOCH_3_, none of the studied pathways would be thermochemically feasible).

### 3.3. Comparison to Other Studies

Matxain et al. reported a DFT study on the reactivity of PN with •OH, •OOH and •O_2_${}^{-}$ [28]. The study concluded, from thermodynamical considerations, that •OH would preferentially abstract a hydrogen atom from any of the methylene moieties or from the phenol group ($\Delta G^{0}$ between −33 and −39 kcal/mol), while it could also form adducts at the aromatic C atoms adjacent to the pyridine nitrogen (i.e., C2 and C6) ($\Delta G^{0}$ around −6 kcal/mol). On the other hand, •OOH would only react at the same two aromatic C atoms ($\Delta G^{0}$ around −7 kcal/mol) and •O_2_${}^{-}$ would not react at all. Despite focusing on a different vitamin B_6_ vitamer and using a different DFT functional (B3LYP), these results are in qualitative agreement with our kinetic study, which shows that, in the case of PM, the phenol hydrogen abstraction is among the preferred routes for the more reactive •OCH_3_ radical ($k_{overall}$≈ 10^9^ M${}^{-1}$ s${}^{-1}$) with hydrogen abstractions from the methylene groups (C8 and C9) being also highly favorable ($k_{overall}$≈ 10^4^–10^6^ M${}^{-1}$ s${}^{-1}$), while the less reactive •OOH and •OOCH_3_ radicals would be preferentially scavenged via additions at C2 and/or C6 ($k_{overall}$≈ 10^2^–10^4^ M${}^{-1}$ s${}^{-1}$). The difference is that, unlike the PN model used in the study by Matxain et al., PM displays additional tautomers containing a protonated pyridine nitrogen or a protonated amino group that also represent preferred HAT reaction sites.

The differences in thermodynamics and kinetics among the three studied radicals are in line with their different stabilities: peroxyl radicals ROO• are much more stable than corresponding alkoxyl radicals RO• due to electron donation from the adjacent oxygen lone pairs to the half-empty orbital on the terminal oxygen (hence, •OOCH_3_ is much more stable than •OCH_3_), and the methyl group in •OOCH_3_ also makes it slightly more stable than •OOH due to electron density donation. The more stable a radical is, the less reactivity it will show, hence •OOCH_3_ is much less reactive than •OCH_3_ and slightly less reactive than •OOH. Identical reactivity trends are obtained in many similar DFT studies of antioxidant-free radical reactions [31,32,58], including a recent investigation on the thermodynamics of HAT reactions between cinnamic acid derivatives and ten oxygen-centered radicals of different nature, in both water and pentyl ethanoate [59].

Considering that •OOH and •OOCH_3_ are less reactive than other free radicals, overall rate constants in water in the order of 10^4^ and 10^3^ M${}^{-1}$ s${}^{-1}$, respectively, for their reactions with PM indicate a notorious radical scavenging ability. The overall rate constant for •OOH is similar to that of antioxidants such as capsaicin (2.07 × 10^4^ M${}^{-1}$ s${}^{-1}$) [58] or β-carotene (5.69 × 10^4^ M${}^{-1}$ s${}^{-1}$) [60] and better than that of allicin (8 × 10^3^ M${}^{-1}$ s${}^{-1}$) [61] or melatonin (2 × 10^1^ M${}^{-1}$ s${}^{-1}$) [34]. However, it does not perform as well as α-tocopherol (1.5 × 10^5^–7.9 × 10^6^ M${}^{-1}$ s${}^{-1}$) [62], canolol (2.47 × 10^6^ M${}^{-1}$ s${}^{-1}$) [33], most guaiacol derivatives (1.54 × 10^5^–1.65 × 10^7^ M${}^{-1}$ s${}^{-1}$) [63] or sesamol (2.44 × 10^8^ M${}^{-1}$ s${}^{-1}$) [64].

## 4. Discussion

Although the active form of vitamin B_6_ in our bodies is pyridoxal 5’-phosphate (PLP) and in food it is mainly present as PN, PM has been regarded as the vitamin B_6_ vitamer with the highest ability to inhibit post-Amadori reactions and, consequently, the formation of AGEs [23,65]. For this reason, the present study focuses specifically on this form of vitamin B_6_ to shed new light on one of the possible mechanisms underlying this inhibitory activity. The other two forms have also been reported to show antioxidant activity [66,67] and the aforementioned study on the ROS trapping ability of PN [28] shows that it shares some of the preferred reaction sites with PM (namely, the phenol group, the methylene moieties and the aromatic carbons adjacent to the pyridine nitrogen). Since PLP also displays all these structural elements, it is plausible that it also scavenges ROS by similar mechanisms.

PM has been proven to act therapeutically on diabetic rats and humans displaying renal, retinal or cardiovascular dysfunctions [12,13,14,15]. Several attempts to elucidate its mechanism of action have pointed PM as a potent inhibitor of post-Amadori reactions, both in vitro and in vivo [23]. Over the past decade, our group has studied the reactivity of PM towards several carbohydrates and other carbonyls with a notorious glycation potential. Our results revealed that PM can form Schiff bases with rate constants one order of magnitude higher than those for the same reactions occurred on amino acids [19,38]. These results suggest that PM is also an effective inhibitor of the first stages of the protein glycation process.

Our group has also conducted studies on the ability of PM to trap different model Amadori compounds and to chelate different metal cations which catalyze their autoxidation [16]. These studies concluded that the key to explain the inhibitory activity of PM is its strong metal chelation ability and not its interaction with Amadori intermediates, in agreement with a previous work by Voziyan and colleagues [68]. This conclusion was further supported by two DFT studies showing that PM could compete against a model Amadori compound to chelate Cu^2+^ and Fe^3+^, while aminoguanidine could not, thereby explaining the fact that aminoguanidine cannot prevent the oxidation of Amadori compounds despite its ability to chelate metal ions [35,36]. Although PM does not interact directly with the carbonyl group of Amadori compounds, it can inhibit its degradation to many small RCS such as glyoxal, methylglyoxal and glycolaldehyde [20,69,70].

In the 2000s, several works additionally suggested a potent antioxidant activity of vitamin B_6_, comparable to that of α-tocopherol or ascorbic acid [71,72,73,74,75]. In fact, PM is able to scavenge the ABTS^+^• radical cation used in the TEAC assay, although relatively slowly [76]. Subsequent theoretical studies carried out by Matxain and coworkers showed that PN, another form of vitamin B_6_, is highly reactive towards singlet oxygen [77] and hydroxyl radicals [28,29] but not against superoxide [28]. The transfer of the phenolic hydrogen atom could contribute significantly to this antioxidant activity [78], in perfect agreement with our findings. Furthermore, a recent study showing that PM reduces the levels of intracellular ROS induced by the glycation of the human serum albumin protein, further supports the idea that PM is a powerful antioxidant [79].

The study reported herein strengthens the hypothesis that PM can inhibit protein glycation and the cellular damage induced by its side reactions through the efficient scavenging of ROS. We have proved that PM would efficiently trap reactive radical species such as •OCH_3_ and, in an aqueous environment, it would be moderately reactive towards peroxyl (•OOH) or alkyl peroxyl radicals such as •OOCH_3_. This ability of PM to trap ROS would complement its activity as a metal chelator and as a small RCS scavenger in the context of post-Amadori inhibition of AGEs formation.

Two limitations of this study should be pointed out. The first one is that only the monocationic tautomers of PM have been modeled in water, since more than 99% of PM exists in this form at physiological pH. However, if any of the dicationic, neutral or monoanionic tautomers were reactive enough, this fact would compensate its low mole fraction and it would have a non-negligible role in ROS scavenging. The second one is that only the three most common mechanisms (HAT, RAF and SET) for antioxidant-free radical reactions have been considered, but alternative mechanisms might also contribute. In any case, in either of these two scenarios the conclusion that PM is a potent ROS scavenger would still hold. An interesting follow-up of this study would be the modeling of ROS scavenging by PLP, with the same methodology used herein, to compare the results with PM and find out whether it could act in a similar way in the context of AGE formation inhibition.

## 5. Conclusions

In this study, the primary antioxidant activity of PM, one of the natural forms of vitamin B_6_, together with PN and PL, was investigated by performing DFT calculations at the M05-2X/6-311+G(d,p)/SMD level of theory. The results show that PM is an effective scavenger of the •OCH_3_ radical, in both aqueous ($k_{tot}$ = 5.3 × 10^9^ M${}^{-1}$ s${}^{-1}$) and lipidic ($k_{tot}$ = 3.0 × 10^9^ M${}^{-1}$ s${}^{-1}$) microenvironments, and a moderate scavenger of •OOH and •OOCH_3_ in aqueous media ($k_{tot}$ = 3.2 × 10^4^ and 8.3 × 10^3^ M${}^{-1}$ s${}^{-1}$, respectively), but not in a lipidic media ($k_{tot}$ = 4.2 × 10^1^ and ≈0 M${}^{-1}$ s${}^{-1}$, respectively). From the kinetic point of view, the preferred mechanisms to trap the •OCH_3_ radical are the diffusion-controlled transfer of the hydrogen atoms from the protonated pyridine, from the protonated amino group, and from the phenolic oxygen atom. Hydrogen-atom transfer reactions from the methylene groups in the –CH_2_NH_3_ and the –CH_2_OH ring substituents are also favorable pathways (rate constants in the order of 10^4^–10^6^ M${}^{-1}$ s${}^{-1}$). On the other hand, the formation of radical adducts on the aromatic carbon atoms adjacent to the pyridine nitrogen atom would be preferred when scavenging the •OOH and •OOCH_3_ radicals. These results add to our previous studies on the mechanisms by which PM inhibits post-Amadori reactions in the context of protein glycation by showing that, besides its strength as a metal chelator and its reactivity towards small carbohydrates and reactive carbonyl species, its ability to scavenge reactive oxygen species also plays a significant role.

## Figures and Tables

**Table 1:** Standard reaction Gibbs free energies $\Delta G^{0}$ (in kcal/mol), at 298.15 K and 1 bar, for each combination of reaction site, pyridoxamine tautomer in water and free radical. See Figure 1 for nomenclature.

	H_2_PM(±)			H_2_PM(+)			H_2_PM(0)		
	•OOH	•OOCH_3_	•OCH_3_	•OOH	•OOCH_3_	•OCH_3_	•OOH	•OOCH_3_	•OCH_3_
**HAT-C6**	33.9	34.5	15.4	34.0	34.6	15.5	23.7	24.3	5.2
**HAT-C7**	−2.1	−1.5	−20.6	2.2	2.8	−16.3	4.6	5.2	−13.9
**HAT-C8**	−0.7	−0.1	−19.2	−12.7	−12.1	−31.2	8.7	9.3	−9.8
**HAT-C9**	−1.3	−0.7	−19.8	−1.8	−1.2	−20.3	3.3	3.9	−15.2
**HAT-N1**	−1.4	−0.8	−19.9	26.5	27.1	8.0	-	-	−
**HAT-N2**	10.1	10.6	−8.4	13.5	14.1	−5.0	26.7	27.3	8.2
**HAT-O1**	-	-	-	25.4	12.1	−7.0	13.9	0.6	−18.5
**HAT-O2**	29.8	30.4	11.3	19.0	19.6	0.5	19.4	20.0	0.9
**RAF-N1**	-	-	-	-	-	-	42.7	-	27.6
**RAF-C2**	−5.3	−2.9	−20.5	11.4	15.3	−4.3	12.8	16.2	−1.0
**RAF-C3**	20.7	22.2	4.1	17.9	19.5	2.0	15.2	17.3	0.2
**RAF-C4**	0.9	2.8	−15.4	13.3	16.8	−2.9	17.6	20.3	0.7
**RAF-C5**	19.9	21.5	4.1	19.6	19.8	3.1	16.2	18.2	1.8
**RAF-C6**	−2.5	0.4	−17.4	11.8	13.1	−2.6	10.5	13.6	−3.7
**SET**	26.3	28.1	20.8	62.0	63.8	56.5	41.5	43.3	36.0

HAT: Hydrogen-atom transfer. RAF: Radical-adduct formation. SET: Single-electron transfer. The letter and number next to each abbreviation indicate the reaction site, as defined in Figure 1.

**Table 2:** Standard reaction Gibbs free energies $\Delta G^{0}$ (in kcal/mol), at 298.15 K and 1 bar, for each combination of reaction site and free radical in pentyl ethanoate. See Figure 1 for nomenclature.

	HPM(0)		
	•OOH	•OOCH_3_	•OCH_3_
**HAT-C6**	23.1	24.6	4.8
**HAT-C7**	5.6	7.0	−12.8
**HAT-C8**	−0.2	1.2	−18.5
**HAT-C9**	0.3	1.7	−18.0
**HAT-N2**	19.2	20.6	0.9
**HAT-O1**	6.9	8.3	−11.4
**HAT-O2**	19.7	21.1	1.3
**RAF-N1**	42.6	-	29.3
**RAF-C2**	11.1	15.0	−1.8
**RAF-C3**	14.8	19.3	0.0
**RAF-C4**	13.8	17.2	−1.8
**RAF-C5**	16.1	18.8	1.1
**RAF-C6**	10.7	15.6	−3.6
**SET**	89.4	90.7	81.3

HAT: Hydrogen-atom transfer. RAF: Radical-adduct formation. SET: Single-electron transfer. The letter and number next to each abbreviation indicate the reaction site, as defined in Figure 1.

**Table 3:** Rate constants *k* (in M${}^{-1}$ s${}^{-1}$), at 298.15 K and 1 bar, for those combinations of reaction site, pyridoxamine tautomer and free radical where the standard reaction Gibbs free energy is less than 1 kcal/mol. See Figure 1 for nomenclature.

	H_2_PM(±)			H_2_PM(+)			H_2_PM(0)	HPM(0)	
	•OOH	•OOCH_3_	•OCH_3_	•OOH	•OOCH_3_	•OCH_3_	•OCH_3_	•OOH	•OCH_3_
**HAT-C7**	2.8 × 10^1^	2.8 × 10^0^	3.3 × 10^5^	-	-	1.4 × 10^5^	1.0 × 10^5^	-	2.0 × 10^4^
**HAT-C8**	1.1 × 10^0^	1.1 × 10^−1^	4.2 × 10^4^	1.8 × 10^0^	2.7 × 10^0^	9.6 × 10^4^	4.2 × 10^3^	4.0 × 10^1^	1.7 × 10^6^
**HAT-C9**	6.8 × 10^−1^	2.8 × 10^−1^	7.0 × 10^5^	2.9 × 10^0^	1.8 × 10^−1^	3.1 × 10^5^	1.8 × 10^5^	1.1 × 10^0^	7.3 × 10^4^
**HAT-N1**	-	-	2.9 × 10^9^	-	-	-	-	-	-
**HAT-N2**	-	-	2.8 × 10^9^	-	-	5.6 × 10^1^	-	-	1.7 × 10^2^
**HAT-O1**	-	-	-	-	-	3.0 × 10^9^	2.9 × 10^9^	-	3.0 × 10^9^
**HAT-O2**	-	-	-	-	-	2.8 × 10^−1^	5.6 × 10^-2^	-	-
**RAF-C2**	3.1 × 10^4^	1.1 × 10^4^	9.1 × 10^7^	-	-	6.1 × 10^−1^	3.9 × 10^0^	-	1.1 × 10^2^
**RAF-C3**	-	-	-	-	-	-	1.2 × 10^1^	-	3.5 × 10^3^
**RAF-C4**	1.1 × 10^2^	-	6.4 × 10^6^	-	-	3.8 × 10^4^	1.6 × 10^0^	-	9.5 × 10^3^
**RAF-C6**	9.9 × 10^3^	2.1 × 10^2^	2.1 × 10^8^	-	-	1.8 × 10^0^	2.8 × 10^3^	-	1.5 × 10^4^

HAT: Hydrogen-atom transfer. RAF: Radical-adduct formation. The letter and number next to each abbreviation indicate the reaction site, as defined in Figure 1.

**Table 4:** Overall rate constants $k_{overall}$ (in M${}^{-1}$ s${}^{-1}$) and branching ratios, at 298.15 K and 1 bar, and at physiological pH (7.4), for each free radical in each solvent at each reaction site. Total rate constants $k_{tot}$ (in M${}^{-1}$ s${}^{-1}$), calculated as the sum of all $k_{overall}$ for each radical in each solvent, are also indicated. See Figure 1 for nomenclature.

	Water			Pentyl Ethanoate	
	•OOH	•OOCH_3_	•OCH_3_	•OOH	•OCH_3_
**HAT-C7**	2.1 × 10^1^ (<0.1%)	2.1 × 10^0^ (<0.1%)	2.8 × 10^5^ (<0.1%)	≈0	2.0 × 10^4^ (<0.1%)
**HAT-C8**	1.1 × 10^0^ (<0.1%)	4.1 × 10^−1^ (<0.1%)	4.4 × 10^4^ (<0.1%)	4.0 × 10^1^ (97.3%)	1.7 × 10^6^ (<0.1%)
**HAT-C9**	8.7 × 10^−1^ (<0.1%)	2.4 × 10^−1^ (<0.1%)	6.0 × 10^5^ (<0.1%)	1.1 × 10^0^ (2.7%)	7.3 × 10^4^ (<0.1%)
**HAT-N1**	≈0	≈0	2.2 × 10^9^ (42.0%)	-	-
**HAT-N2**	≈0	≈0	2.2 × 10^9^ (41.2%)	≈0	1.7 × 10^2^ (<0.1%)
**HAT-O1**	≈0	≈0	6.6 × 10^8^ (12.4%)	≈0	3.0 × 10^9^ (99.9%)
**HAT-O2**	≈0	≈0	3.9 × 10^-2^ (<0.1%)	≈0	≈0
	**•OOH**	**•OOCH_3_**	**•OCH_3_**	**•OOH**	**•OCH_3_**
**RAF-C2**	2.4 × 10^4^ (75.6%)	8.1 × 10^3^ (98.0%)	6.9 × 10^7^ (1.3%)	≈0	1.1 × 10^2^ (<0.1%)
**RAF-C3**	≈0	≈0	1.2 × 10^0^ (<0.1%)	≈0	3.5 × 10^3^ (<0.1%)
**RAF-C4**	8.6 × 10^1^ (0.3%)	≈0	4.9 × 10^6^ (0.1%)	≈0	9.5 × 10^3^ (<0.1%)
**RAF-C6**	7.6 × 10^3^ (24.0%)	1.6 × 10^2^ (1.9%)	1.6 × 10^8^ (3.1%)	≈0	1.5 × 10^4^ (<0.1%)
$k_{\mathrm{tot}}$	3.2 × 10^4^	8.3 × 10^3^	5.3 × 10^9^	4.2 × 10^1^	3.0 × 10^9^

HAT: Hydrogen-atom transfer. RAF: Radical-adduct formation. The letter and number next to each abbreviation indicate the reaction site, as defined in Figure 1.

## Supplementary Materials

The following are available online at https://www.mdpi.com/2076-3921/8/9/344/s1, Table S1 : Imaginary frequencies and κ values for TSs in water, Table S2: Imaginary frequencies and κ values for TSs in pentyl ethanoate, Table S3: Gibbs free energies of activation for reactions in water, TableS4: Gibbs free energies of activation for reactions in pentyl ethanoate, Cartesian coordinates and figures of all located TSs.

## Abbreviations

The following abbreviations are used in this manuscript:

AGE	Advanced glycation end product
ROS	Reactive oxygen species
RCS	Reactive carbonyl species
NMR	Nuclear magnetic resonance
DFT	Density functional theory
PM	Pyridoxamine
PL	Pyridoxal
PN	Pyridoxine
PLP	Pyridoxal 5’-phosphate
QM-ORSA	Quantum mechanics-based test for overall free radical scavenging activity
TS	Transition state
SMD	Solvation Model based on the quantum mechanical charge Density of a solute molecule interacting with a continuum
HAT	Hydrogen atom transfer
RAF	Radical adduct formation
SET	Single electron transfer
TST	Transition state theory
