# Supplementary material for: How Does Pyridoxamine Inhibit the Formation of Advanced Glycation End Products? The Role of Its Primary Antioxidant Activity
Source: Antioxidants (Basel). 2019 Sep 1;8(9):344. doi: 10.3390/antiox8090344 (PMC6770850; doi:10.3390/antiox8090344)

Supplementary Materials: How Does Pyridoxamine Inhibit the Formation of Advanced Glycation End Products? The Role of Its Primary Antioxidant Activity

Rafael Ramis [1,2], Joaquín Ortega-Castro [1,2,*], Carmen Caballero [1,2], Rodrigo Casasnovas [1,2], Antonia Cerrillo [1,2], Bartolomé Vilanova [1,2], Miquel Adrover [1,2] and Juan Frau [1,2]

Supporting Information
(50 pages)

Contents:

Table S1: Imaginary frequencies (in cm^{-1}) for each TS corresponding to a combination of reaction site, pyridoxamine tautomer in water and free radical where the standard reaction Gibbs free energy is less than 1 kcal/mol. For HAT reactions, tunneling corrections κ are given in parentheses.

Table S2: Imaginary frequencies (in cm^{-1}) for each TS corresponding to a combination of reaction site and free radical in pentyl ethanoate where the standard reaction Gibbs free energy is less than 1 kcal/mol. For HAT reactions, tunneling corrections κ are given in parentheses.

Table S3: Activation Gibbs free energies $\Delta G^{\neq}$ (in kcal/mol), at 298.15 K and 1 bar, for those combinations of reaction site, pyridoxamine tautomer in water and free radical where the standard reaction Gibbs free energy is less than 1 kcal/mol.

Table S4: Activation Gibbs free energies $\Delta G^{\neq}$ (in kcal/mol), at 298.15 K and 1 bar, for those combinations of reaction site and free radical in pentyl ethanoate where the standard reaction Gibbs free energy is less than 1 kcal/mol.

Cartesian coordinates and structures of the optimized geometries in water of the located transition states. In the pictures, distances are given in Å.

Cartesian coordinates and structures of the optimized geometries in pentyl ethanoate of the located transition states. In the pictures, distances are given in Å.

Table S1. Imaginary frequencies (in cm^{-1}) for each TS corresponding to a combination of reaction site, pyridoxamine tautomer in water and free radical where the standard reaction Gibbs free energy is less than 1 kcal/mol. For HAT reactions, tunneling corrections κ are given in parentheses. See Figure 1 for nomenclature.

	H_2PM(±)			H_2PM(+)			H_2PM(0)
	•OOH	•$OOCH_3$	•OCH_3	•OOH	•$OOCH_3$	•OCH_3	•OCH_3
**HAT-C7**	1853.0 (105.6)	2243.8 (1423.5)	1783.4 (23.7)	-	-	1700.8 (19.8)	1594.4 (13.4)
**HAT-C8**	2129.1 (920.3)	2256.2 (1747.4)	1893.4 (46.8)	1899.0 (270.9)	1878.6 (172.6)	1663.6 (15.8)	1852.3 (715.8)
**HAT-C9**	1841.3 (134.2)	1891.2 (132.4)	1475.8 (7.9)	1811.7 (87.1)	2033.0 (418.7)	1603.0 (13.7)	1396.9 (6.9)
**HAT-N2**	-	-	-	-	-	1763.7 (60.3)	-
**HAT-O2**	-	-	-	-	-	23.7 (10022.4)	684.8 (1.7)
**RAF-C2**	471.9	487.8	388.1	-	-	566.5	468.0
**RAF-C3**	-	-	-	-	-	-	534.3
**RAF-C4**	460.0	-	385.1	-	-	626.6	529.3
**RAF-C6**	459.2	475.4	424.9	-	-	566.7	475.2

HAT: Hydrogen-atom transfer. RAF: Radical-adduct formation. The letter and number next to each abbreviation indicate the reaction site, as defined in Figure 1.

Table S2. Imaginary frequencies (in cm^{-1}) for each TS corresponding to a combination of reaction site and free radical in pentyl ethanoate where the standard reaction Gibbs free energy is less than 1 kcal/mol. For HAT reactions, tunneling corrections κ are given in parentheses. See Figure 1 for nomenclature.

	HPM(0)	
	•OOH	•OCH_3
**HAT-C7**	-	1553.8 (15.5)
**HAT-C8**	1865.3 (64.7)	1509.3 (8.4)
**HAT-C9**	1608.7 (29.2)	1520.8 (11.8)
**HAT-N2**	-	1340.5 (7.3)
**RAF-C2**	-	461.3
**RAF-C3**	-	457.7
**RAF-C4**	-	479.1
**RAF-C6**	-	411.4

HAT: Hydrogen-atom transfer. RAF: Radical-adduct formation. The letter and number next to each abbreviation indicate the reaction site, as defined in Figure 1.

Table S3. Activation Gibbs free energies $\Delta G^{\neq}$ (in kcal/mol), at 298.15 K and 1 bar, for those combinations of reaction site, pyridoxamine tautomer in water and free radical where the standard reaction Gibbs free energy is less than 1 kcal/mol. See Figure 1 for nomenclature.

	**H_2PM(±)**			**H_2PM(+)**			**H_2PM(0)**
	**•OOH**	**•$OOCH_3$**	**•OCH_3**	**•OOH**	**•$OOCH_3$**	**•OCH_3**	**•OCH_3**
**HAT-C7**	18.9	15.3	12.5	-	-	12.9	12.8
**HAT-C8**	21.8	23.6	13.8	20.8	20.3	12.7	16.8
**HAT-C9**	21.0	21.5	11.1	19.9	22.5	11.9	11.8
**HAT-N2**	-	-	-	-	-	17.9	-
**HAT-O2**	-	-	-	-	-	-	19.5
**RAF-C2**	11.7	12.4	7.0	-	-	18.1	17.1
**RAF-C3**	-	-	-	-	-	-	16.4
**RAF-C4**	15.0	-	8.6	-	-	11.6	17.6
**RAF-C6**	12.4	14.7	6.4	-	-	17.5	13.2

HAT: Hydrogen-atom transfer. RAF: Radical-adduct formation. The letter and number next to each abbreviation indicate the reaction site, as defined in Figure 1.

Table S4. Activation Gibbs free energies $\Delta G^{\neq}$ (in kcal/mol), at 298.15 K and 1 bar, for those combinations of reaction site and free radical in pentyl ethanoate where the standard reaction Gibbs free energy is less than 1 kcal/mol. See Figure 1 for nomenclature.

	**HPM(0)**	
	**•OOH**	**•OCH_3**
**HAT-C7**	-	13.9
**HAT-C8**	18.1	10.6
**HAT-C9**	19.8	12.7
**HAT-N2**	-	16.0
**RAF-C2**	-	15.1
**RAF-C3**	-	13.0
**RAF-C4**	-	12.4
**RAF-C6**	-	12.2

HAT: Hydrogen-atom transfer. RAF: Radical-adduct formation. The letter and number next to each abbreviation indicate the reaction site, as defined in Figure 1.

Cartesian coordinates and structures of the optimized geometries in water of the located transition states. In the pictures, distances are given in Å.

TS-H_2PM(±)(-H,C7)(OOH)$^{+\bullet}$

Charge=1, Multiplicity=2

C	0.523855	0.521441	-0.848941
C	-0.863422	0.273661	-0.616952
C	-1.327021	-0.992796	-0.289813
C	-0.422973	-2.043889	-0.233660
N	0.868884	-1.821641	-0.491768
H	-0.703689	-3.057238	0.006137
C	-1.761352	1.467979	-0.722716
H	-1.616178	1.961325	-1.680830
H	-2.808249	1.228569	-0.589286
C	-2.770384	-1.259958	0.054927
H	-3.426008	-0.917531	-0.742188
H	-2.919589	-2.330705	0.187212
O	0.970595	1.686571	-1.071610
O	-3.159924	-0.549866	1.232079
H	-2.673059	-0.916215	1.977774
N	-1.383078	2.464057	0.327973
H	-1.937443	3.316131	0.240565
H	-1.523492	2.076785	1.261636
C	1.399526	-0.618369	-0.795571
C	2.817915	-0.478563	-0.945176
H	3.121424	0.334788	-1.594948
H	3.158765	-0.063021	0.184773
H	3.366513	-1.404861	-1.086247
H	-0.391787	2.697709	0.208008
H	1.505406	-2.612093	-0.447222
O	3.199054	0.356056	1.449086
O	1.881620	0.324597	1.819863
H	1.739115	-0.566461	2.173243

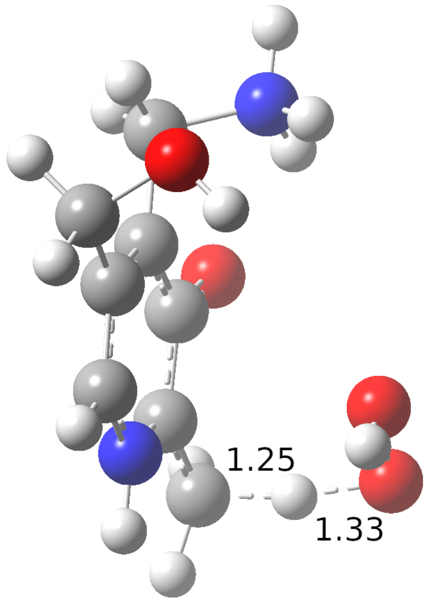

TS-H_2PM(±)(-H,C8)(OOH)$^{+\bullet}$

```
Charge=1, Multiplicity=2
C   -0.770781   -1.038078    0.237429
C   -0.078233    0.205627    0.153524
C   -0.746756    1.367050   -0.290023
C   -2.077147    1.295786   -0.594409
N   -2.725266    0.110991   -0.480056
H   -2.664883    2.135953   -0.927162
C    1.352193    0.244795    0.425237
H    1.925462   -0.189244   -0.621027
H    1.798933    1.218441    0.578445
C   -0.044452    2.695186   -0.411083
H    0.870030    2.590003   -0.990841
H   -0.698256    3.399577   -0.923659
O   -0.209231   -2.138016    0.573249
O    0.343032    3.209517    0.864131
H   -0.457509    3.391769    1.366757
N    1.827175   -0.711612    1.444501
H    2.812951   -0.937829    1.286964
H    1.727216   -0.326596    2.385159
C   -2.163732   -1.026212   -0.088746
C   -2.967467   -2.269690   -0.015472
H   -2.555798   -3.010503   -0.702439
H   -2.902671   -2.685906    0.990315
H   -4.008786   -2.081369   -0.266555
H    1.243310   -1.569254    1.345957
H   -3.713297    0.090682   -0.716461
O    2.707702   -0.806822   -1.411991
O    3.843127   -1.061886   -0.679240
H    4.411141   -0.290514   -0.824489
```

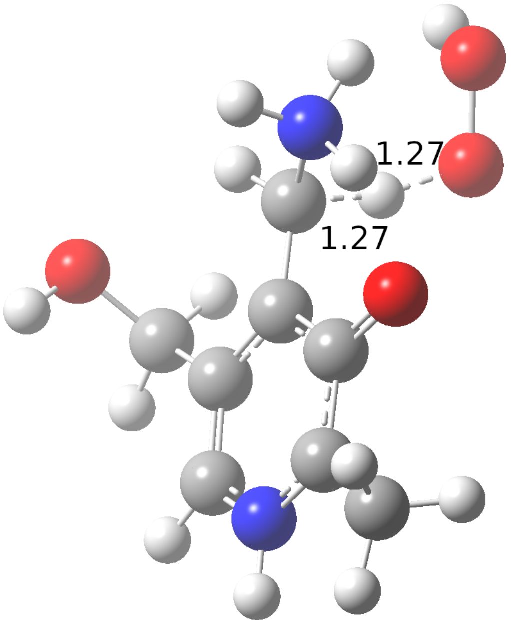

TS-H_2PM(+)(-H,C8)(OOH)$^{+\bullet}$

```
Charge=1, Multiplicity=2
C    -0.069015    -1.036162     0.298184
C    -0.150784     0.347834     0.102320
C    -1.356541     0.896700    -0.369552
C    -2.414510     0.056647    -0.612171
N    -2.284180    -1.264923    -0.404568
H    -3.375344     0.396417    -0.965658
C     1.058862     1.169467     0.318727
H     1.794784     0.821972    -0.644691
H     0.946378     2.232862     0.130096
C    -1.537548     2.379629    -0.572454
H    -0.755966     2.777526    -1.214722
H    -2.499987     2.559028    -1.050061
O     1.060163    -1.655721     0.700094
O    -1.449460     3.086621     0.663541
H    -2.200604     2.831499     1.209622
N     1.803184     0.807270     1.481597
H     2.586121     1.428371     1.631591
H     1.226880     0.765728     2.316403
C    -1.168695    -1.853516     0.041150
C    -1.128253    -3.325612     0.232122
H    -0.382377    -3.762195    -0.432612
H    -0.835673    -3.552725     1.256994
H    -2.099796    -3.768106     0.024748
H    -3.087210    -1.858807    -0.593192
H     1.679524    -0.970196     1.046051
O     2.661000     0.319782    -1.466953
O     3.671546    -0.176999    -0.692602
H     3.423145    -1.097200    -0.516380
```

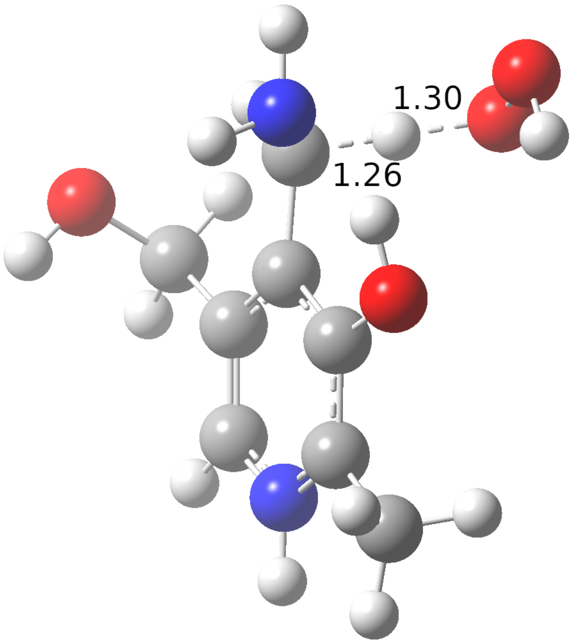

TS-H_2PM(±)(-H,C9)(OOH)$^{+\bullet}$

Charge=1, Multiplicity=2

C	1.933861	-0.099879	-0.119870
C	0.741304	0.635549	-0.332572
C	-0.505630	0.001381	-0.359859
C	-0.570919	-1.361306	-0.178622
N	0.573129	-2.042634	0.019496
H	-1.483697	-1.933309	-0.184461
C	0.899706	2.110103	-0.557175
H	1.480727	2.288447	-1.460062
H	-0.043514	2.637627	-0.624361
C	-1.768607	0.785141	-0.553600
H	-1.810285	1.384957	-1.458494
H	-2.711317	-0.052940	-0.731924
O	3.091951	0.455855	-0.084520
O	-2.144789	1.560893	0.534059
H	-1.974559	1.074780	1.351351
N	1.681125	2.703531	0.570089
H	1.886583	3.686333	0.393919
H	1.170957	2.628909	1.450249
C	1.792534	-1.504807	0.061044
C	2.975840	-2.371650	0.284114
H	3.667936	-2.269243	-0.552644
H	3.502535	-2.047305	1.182607
H	2.688006	-3.415515	0.389754
H	2.559357	2.167638	0.639552
H	0.500178	-3.047398	0.152244
O	-3.734220	-0.785260	-0.601282
O	-3.924807	-0.833140	0.765215
H	-4.506302	-0.083240	0.956636

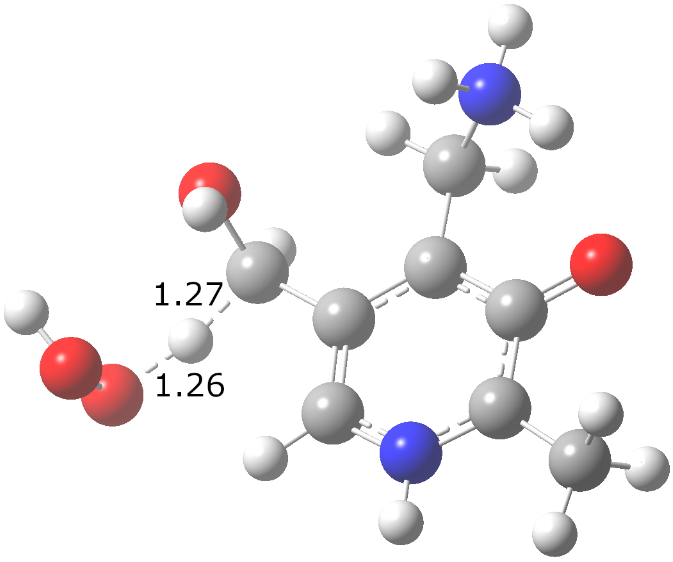

TS-H_2PM(+)(-H,C9)(OOH)$^{+\bullet}$

Charge=1, Multiplicity=2

C	1.658975	0.157479	-0.125956
C	0.401413	0.644682	0.237728
C	-0.592331	-0.269535	0.630760
C	-0.300190	-1.616376	0.634905
N	0.927574	-2.025959	0.282237
H	-0.994639	-2.386761	0.926287
C	0.111082	2.120363	0.134415
H	-0.210652	2.316046	-0.890514
H	-0.710649	2.395904	0.791603
C	-1.961424	0.191499	0.982264
H	-2.005056	0.978728	1.728982
H	-2.375383	0.724110	-0.081986
O	2.649180	0.966453	-0.541123
O	-2.881788	-0.800921	1.282471
H	-2.994925	-1.374557	0.509648
N	1.315395	2.923064	0.399699
H	1.186565	3.857650	0.033780
H	1.464055	3.001499	1.399014
C	1.922936	-1.213863	-0.089960
C	3.244003	-1.778557	-0.463824
H	3.446712	-1.573965	-1.515705
H	4.025234	-1.297949	0.124572
H	3.268170	-2.852498	-0.293646
H	1.119237	-3.023755	0.305715
H	2.350281	1.911428	-0.268888
O	-2.833765	0.739354	-1.292171
O	-2.944243	-0.596783	-1.597955
H	-2.091588	-0.834598	-1.994178

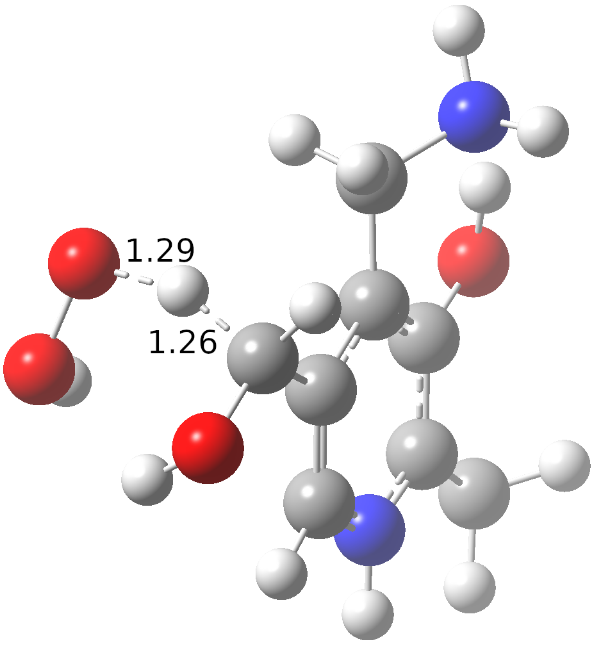

TS-H_2PM(±)(-H,C7)($OOCH_3$)$^{+\bullet}$

Charge=1, Multiplicity=2

C	-0.001429	0.177513	-0.817985
C	-1.389378	0.301382	-0.517756
C	-2.132837	-0.778934	-0.058807
C	-1.513130	-2.011886	0.067607
N	-0.221078	-2.135881	-0.252183
H	-2.018088	-2.900760	0.410876
C	-1.979372	1.664022	-0.718182
H	-1.796357	2.003180	-1.735218
H	-3.039183	1.705915	-0.503900
C	-3.578110	-0.646969	0.350314
H	-4.168782	-0.223631	-0.458513
H	-3.978887	-1.630884	0.590563
O	0.704995	1.174823	-1.172074
O	-3.722595	0.238497	1.462605
H	-3.299803	-0.168592	2.225898
N	-1.291613	2.639651	0.182962
H	-1.634202	3.586771	0.022564
H	-1.446735	2.400004	1.162847
C	0.567137	-1.132873	-0.687607
C	1.964345	-1.378170	-0.925111
H	2.416468	-0.722936	-1.662297
H	2.548479	-0.999193	0.129968
H	2.245555	-2.423244	-1.013091
H	-0.286152	2.605140	-0.019091
H	0.204291	-3.053972	-0.159802
O	3.192378	-0.605751	1.183246
O	4.353850	-0.041591	0.741329
C	4.123205	1.335922	0.428337
H	3.767936	1.857341	1.315100
H	5.087647	1.725222	0.112512
H	3.395031	1.405391	-0.379574

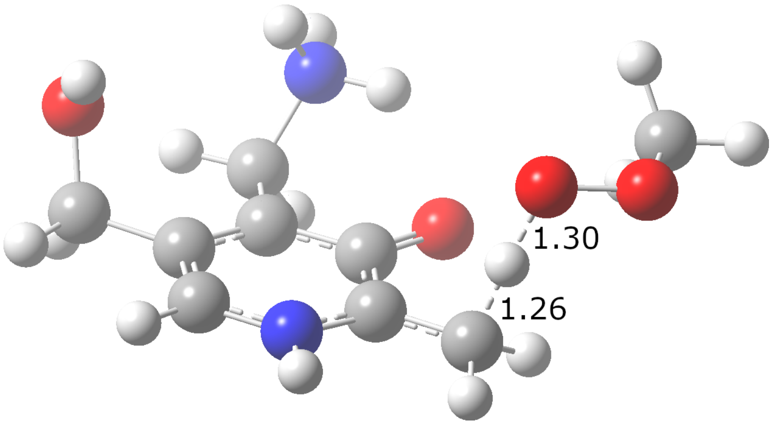

TS-H_2PM(±)(-H,C8a)($OOCH_3$)$^{+\bullet}$

Charge=1, Multiplicity=2

C	0.496744	1.098113	0.420462
C	0.399926	-0.316084	0.255520
C	1.440610	-1.029327	-0.382651
C	2.550000	-0.355972	-0.809405
N	2.629278	0.984662	-0.627200
H	3.390402	-0.825356	-1.294610
C	-0.814158	-1.017085	0.641624
H	-1.613006	-0.953596	-0.368426
H	-0.730409	-2.083743	0.802233
C	1.382004	-2.523628	-0.579417
H	0.441756	-2.814742	-1.043182
H	2.195988	-2.829564	-1.235409
O	-0.421069	1.816923	0.951366
O	1.451385	-3.224832	0.663429
H	2.314371	-3.055435	1.055324
N	-1.601888	-0.385291	1.718532
H	-2.598478	-0.582885	1.605849
H	-1.306284	-0.724518	2.635799
C	1.692777	1.725382	-0.048187
C	1.884761	3.189071	0.085540
H	1.097707	3.712828	-0.458681
H	1.800730	3.474518	1.134597
H	2.856022	3.491600	-0.298753
H	-1.429739	0.645480	1.650039
H	3.463697	1.459225	-0.960229
O	-2.595304	-0.902825	-1.158733
O	-3.558037	-0.159797	-0.528956
C	-3.375154	1.226965	-0.843385
H	-3.436344	1.365475	-1.920754
H	-4.188995	1.742401	-0.339961
H	-2.410009	1.562355	-0.460601

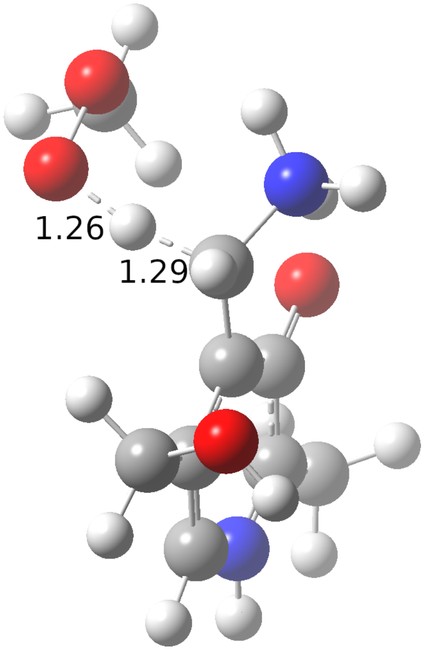

TS-H_2PM(+)(-H,C8)($OOCH_3$)$^{+\bullet}$

Charge=1, Multiplicity=2

C	-1.217302	-0.749019	0.494833
C	-0.208312	0.221242	0.465568
C	-0.435919	1.400134	-0.274869
C	-1.604603	1.536273	-0.977651
N	-2.521054	0.553172	-0.950663
H	-1.844389	2.405187	-1.570315
C	1.084844	0.052862	1.182419
H	1.962848	0.241042	0.296511
H	1.278030	0.860976	1.881657
C	0.547987	2.543134	-0.289863
H	1.543828	2.195207	-0.550933
H	0.232629	3.271704	-1.035375
O	-1.158500	-1.885041	1.221622
O	0.657724	3.156782	0.994135
H	-0.182440	3.580903	1.196816
N	1.352447	-1.227275	1.741509
H	1.625830	-1.907104	1.035681
H	2.071071	-1.180917	2.451406
C	-2.390844	-0.570774	-0.241184
C	-3.479675	-1.579643	-0.248353
H	-3.841587	-1.733899	0.768352
H	-4.301503	-1.250816	-0.879499
H	-3.097674	-2.532768	-0.613813
H	-3.376677	0.679430	-1.484175
H	-0.304729	-1.885434	1.713889
O	2.913216	0.091399	-0.594517
O	3.043041	-1.261619	-0.721068
C	2.176588	-1.731588	-1.765184
H	2.448455	-1.252870	-2.703461
H	2.345282	-2.804100	-1.814750
H	1.140837	-1.515710	-1.503590

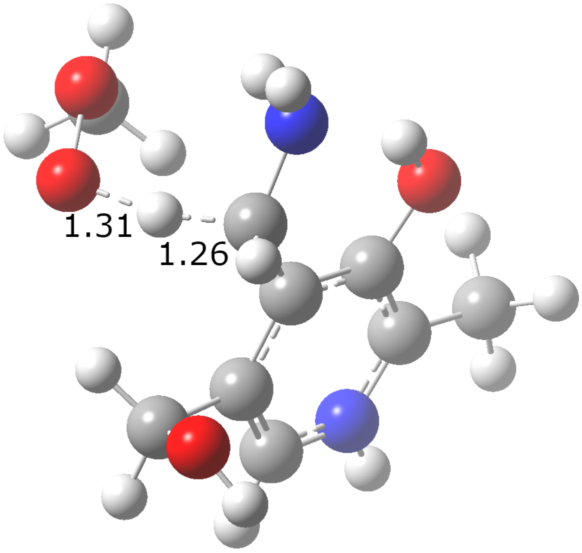

TS-$H_2PM(\pm)(-H,C9)(OOCH_3)^{+\bullet}$

Charge=1, Multiplicity=2

C	2.014563	0.584705	-0.226318
C	0.637057	0.360735	0.020115
C	0.153177	-0.923228	0.295086
C	1.029648	-1.987744	0.282466
N	2.329589	-1.756314	0.027821
H	0.744364	-3.009120	0.473807
C	-0.249519	1.566623	-0.086885
H	-0.181707	1.981338	-1.091048
H	-1.283938	1.367493	0.155534
C	-1.274750	-1.212073	0.582432
H	-1.910829	-0.893344	-0.467482
H	-1.491249	-2.271781	0.700532
O	2.492013	1.754334	-0.460854
O	-1.784179	-0.424355	1.609144
H	-2.656538	-0.756113	1.853210
N	0.247166	2.629537	0.839851
H	-0.278845	3.494382	0.719227
H	0.170360	2.332779	1.812732
C	2.863495	-0.558025	-0.212293
C	4.318331	-0.440339	-0.478622
H	4.477662	-0.024963	-1.474726
H	4.767267	0.249108	0.237295
H	4.810254	-1.408082	-0.407421
H	1.238635	2.789172	0.608992
H	2.957729	-2.555215	0.026681
O	-2.637417	-0.351131	-1.384586
O	-3.505061	0.454341	-0.694528
C	-4.669788	-0.302054	-0.345062
H	-5.308811	0.388221	0.199794
H	-5.163975	-0.652394	-1.249061
H	-4.383790	-1.141162	0.289307

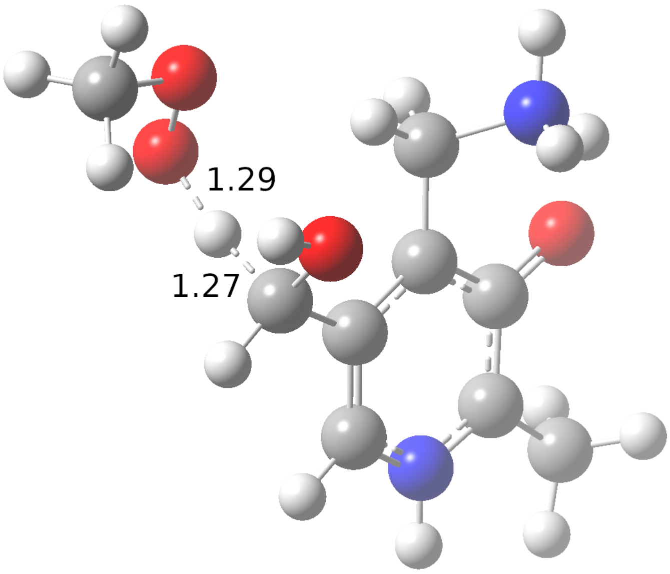

TS-H_2PM(+)(-H,C9)($OOCH_3$)$^{+\bullet}$

Charge=1, Multiplicity=2

C	2.095675	0.340320	-0.223655
C	0.737220	0.468090	0.082022
C	0.016617	-0.682065	0.437253
C	0.654498	-1.902316	0.428009
N	1.953491	-1.969754	0.104109
H	0.168056	-2.832232	0.677084
C	0.085532	1.818967	-0.071768
H	-0.084257	1.965958	-1.140733
H	-0.878469	1.844958	0.427003
C	-1.421085	-0.655960	0.812794
H	-2.047210	-0.146058	-0.163850
H	-1.868105	-1.647695	0.873838
O	2.848354	1.399890	-0.570009
O	-1.658740	0.129626	1.936725
H	-2.577217	0.008812	2.206831
N	0.989359	2.886074	0.389046
H	0.656025	3.779035	0.047953
H	0.986922	2.924830	1.401741
C	2.711088	-0.911550	-0.208986
C	4.143437	-1.100943	-0.547235
H	4.323432	-0.781154	-1.574045
H	4.758554	-0.482132	0.105948
H	4.429856	-2.144266	-0.438527
H	2.397653	-2.884114	0.106177
H	2.315376	2.215003	-0.255549
O	-2.791000	0.349547	-1.084398
O	-3.792886	-0.567997	-1.272186
C	-4.823028	-0.343351	-0.303462
H	-5.210617	0.668210	-0.407550
H	-5.593915	-1.076203	-0.527182
H	-4.421594	-0.502869	0.697908

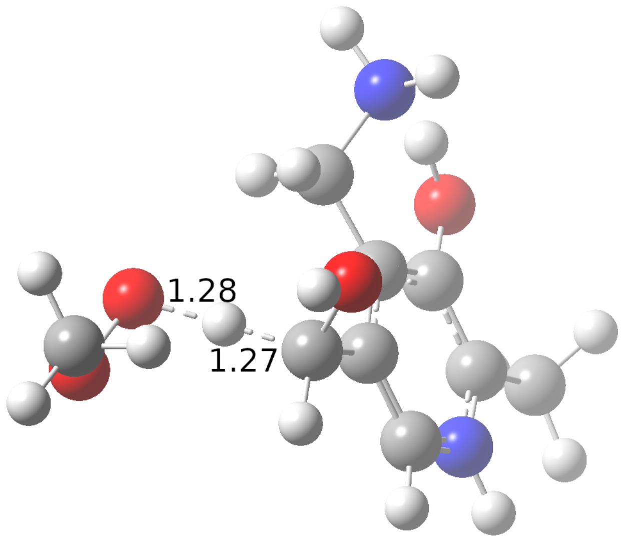

TS-H_2PM(±)(-H,C7)(OCH_3)$^{+•}$

Charge=1, Multiplicity=2

C	0.333725	0.251280	-0.629790
C	-1.078259	0.317492	-0.465327
C	-1.823095	-0.807885	-0.125274
C	-1.172372	-2.018224	0.027995
N	0.152304	-2.079346	-0.160858
H	-1.670068	-2.938524	0.288802
C	-1.701836	1.663009	-0.685324
H	-1.485978	2.012002	-1.693097
H	-2.770803	1.671483	-0.517412
C	-3.311226	-0.751362	0.113303
H	-3.817060	-0.291462	-0.732042
H	-3.696456	-1.763156	0.235271
O	1.040897	1.285200	-0.885737
O	-3.628265	0.045706	1.255728
H	-3.271168	-0.393290	2.034612
N	-1.082416	2.655753	0.245007
H	-1.429326	3.595928	0.057820
H	-1.284460	2.421716	1.217430
C	0.931148	-1.037191	-0.487703
C	2.368348	-1.229580	-0.637166
H	2.789764	-0.635028	-1.444008
H	2.879009	-0.773191	0.333219
H	2.669587	-2.272787	-0.678324
H	-0.065672	2.626938	0.091829
H	0.603226	-2.984190	-0.058954
O	3.618643	-0.052345	1.283278
C	4.529021	0.720729	0.540239
H	5.136904	1.250445	1.283887
H	5.196043	0.097994	-0.058774
H	4.024315	1.462171	-0.081169

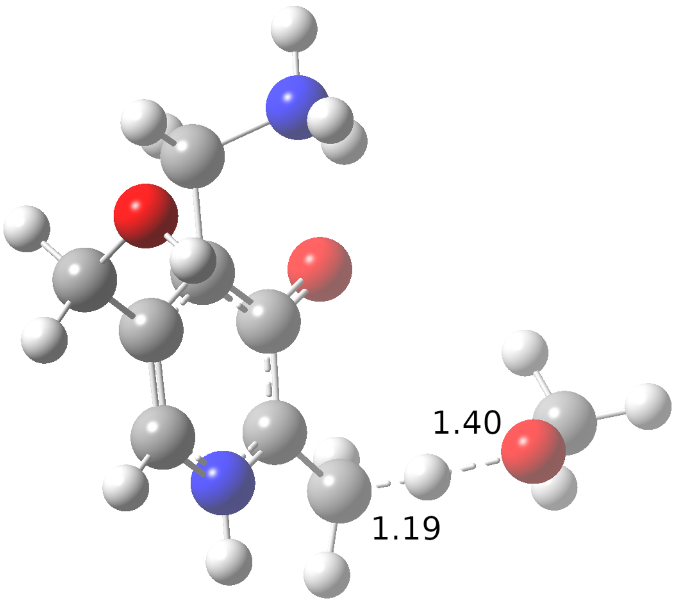

TS-H_2PM(+)(-H,C7)(OCH_3)$^{+\bullet}$

Charge=1, Multiplicity=2

C	0.411735	0.442889	-0.738922
C	-0.957859	0.382559	-0.441864
C	-1.549078	-0.865932	-0.224747
C	-0.768914	-2.000423	-0.312274
N	0.531956	-1.897424	-0.604121
H	-1.153755	-2.997379	-0.163237
C	-1.758355	1.659795	-0.426754
H	-2.012638	1.892413	-1.462957
H	-2.684551	1.525457	0.123665
C	-3.010133	-1.019613	0.118898
H	-3.624326	-0.438770	-0.564387
H	-3.290375	-2.067961	0.026800
O	1.040302	1.604933	-0.956685
O	-3.295264	-0.538581	1.431945
H	-2.854048	-1.113875	2.065322
N	-0.954533	2.774592	0.103663
H	-1.391622	3.653339	-0.143638
H	-0.920726	2.722207	1.115311
C	1.166435	-0.731793	-0.823700
C	2.598505	-0.721495	-1.108685
H	2.875873	-0.000991	-1.872341
H	3.158834	-0.290314	-0.127836
H	3.018099	-1.709328	-1.275064
H	1.077992	-2.751927	-0.673378
H	0.402701	2.324575	-0.580665
O	3.627034	0.194975	1.042374
C	2.590859	0.025898	1.978354
H	3.014415	0.350610	2.936609
H	1.729511	0.660601	1.759081
H	2.296913	-1.020773	2.077024

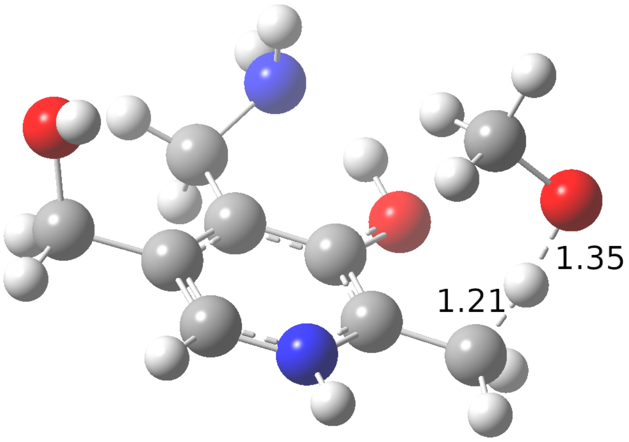

TS-H_2PM(0)(-H,C7)$(OCH_3)^{+\bullet}$

Charge=1, Multiplicity=2

C	-0.528061	-0.133524	-0.873295
C	0.846613	-0.137725	-0.668134
C	1.428198	1.000066	-0.107159
C	0.603888	2.075130	0.202655
N	-0.709790	2.085780	-0.006657
H	1.032398	2.966107	0.643861
C	1.646418	-1.364092	-1.003087
H	1.162114	-1.956808	-1.769798
H	2.651136	-1.111483	-1.326080
C	2.897519	1.059287	0.207335
H	3.493596	0.867259	-0.682132
H	3.151761	2.046179	0.589845
O	-1.099925	-1.258656	-1.378978
O	3.267686	0.049190	1.159990
H	2.921443	0.311119	2.020132
N	1.785257	-2.224288	0.213933
H	2.286919	-3.084639	-0.007577
H	2.315547	-1.697829	0.918671
C	-1.283563	1.006301	-0.545987
C	-2.741966	1.012322	-0.757574
H	-3.061528	0.639292	-1.730435
H	-3.234310	0.223752	0.006844
H	-3.193506	1.968993	-0.517407
H	-2.063093	-1.201979	-1.364801
H	0.869823	-2.470777	0.592951
O	-3.585385	-0.752460	0.905782
C	-2.501402	-0.865307	1.795557
H	-2.869689	-1.480130	2.625686
H	-1.644585	-1.373329	1.347599
H	-2.211155	0.104490	2.205488

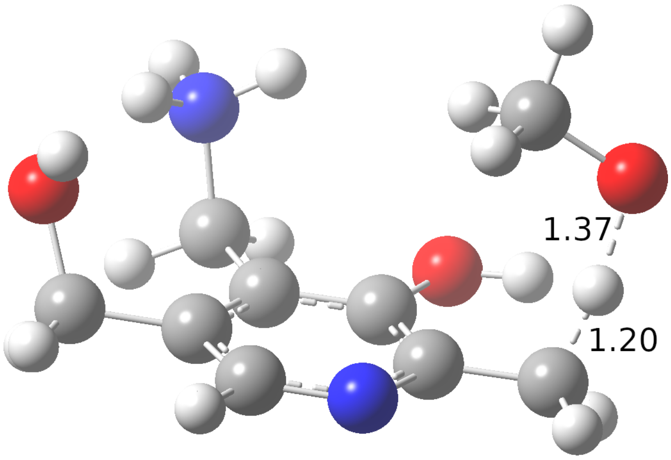

TS-H_2PM(±)(-H,C8)$(OCH_3)^{+\bullet}$

Charge=1, Multiplicity=2

C	-0.952181	-0.984604	0.207011
C	-0.086514	0.141945	0.156116
C	-0.550475	1.391437	-0.291933
C	-1.868080	1.523314	-0.644820
N	-2.683086	0.447632	-0.567946
H	-2.313924	2.444303	-0.983573
C	1.332802	-0.060619	0.493935
H	1.829673	-0.668140	-0.418528
H	1.924888	0.838621	0.607553
C	0.341988	2.605218	-0.357164
H	1.252436	2.384722	-0.909906
H	-0.182947	3.409424	-0.871126
O	-0.563238	-2.153863	0.562350
O	0.751430	3.027684	0.944852
H	-0.031417	3.319272	1.423661
N	1.535337	-0.941759	1.668212
H	2.502655	-1.264308	1.713610
H	1.314670	-0.449012	2.535137
C	-2.311772	-0.765497	-0.172079
C	-3.301636	-1.870008	-0.148299
H	-2.964830	-2.676939	-0.800290
H	-3.375236	-2.275687	0.861492
H	-4.280825	-1.526139	-0.474977
H	0.890231	-1.745541	1.549910
H	-3.655730	0.574117	-0.833351
O	2.675158	-1.368063	-1.202552
C	3.949426	-0.799164	-1.008372
H	3.984367	0.242428	-1.329883
H	4.285143	-0.902754	0.026263
H	4.626945	-1.383679	-1.641438

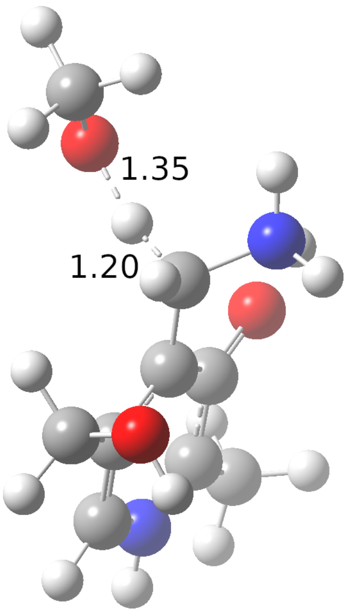

TS-$H_2PM(+)(-H,C8)(OCH_3)^{+\bullet}$

Charge=1, Multiplicity=2

C	-0.327552	-1.049998	0.315970
C	-0.112720	0.323723	0.154468
C	-1.154820	1.121524	-0.347858
C	-2.354345	0.527023	-0.652582
N	-2.508775	-0.797249	-0.482465
H	-3.206256	1.066137	-1.036352
C	1.238195	0.877422	0.431949
H	1.873491	0.544651	-0.529656
H	1.285990	1.961873	0.403836
C	-1.003025	2.608449	-0.550116
H	-0.121307	2.820515	-1.150436
H	-1.876782	2.988041	-1.078241
O	0.630471	-1.886908	0.755585
O	-0.827854	3.294931	0.688103
H	-1.640826	3.209891	1.196551
N	1.875630	0.262324	1.580639
H	2.838407	0.569644	1.647855
H	1.398388	0.544194	2.431968
C	-1.560084	-1.614750	-0.013683
C	-1.833157	-3.067010	0.127486
H	-1.150660	-3.632742	-0.507369
H	-1.658365	-3.373193	1.158801
H	-2.859047	-3.295081	-0.151077
H	-3.405271	-1.209129	-0.726218
H	1.346412	-1.312207	1.160601
O	2.688275	-0.003280	-1.489285
C	3.717440	-0.685297	-0.815744
H	3.339785	-1.528733	-0.236339
H	4.365207	-1.079607	-1.608924
H	4.316130	-0.014252	-0.196781

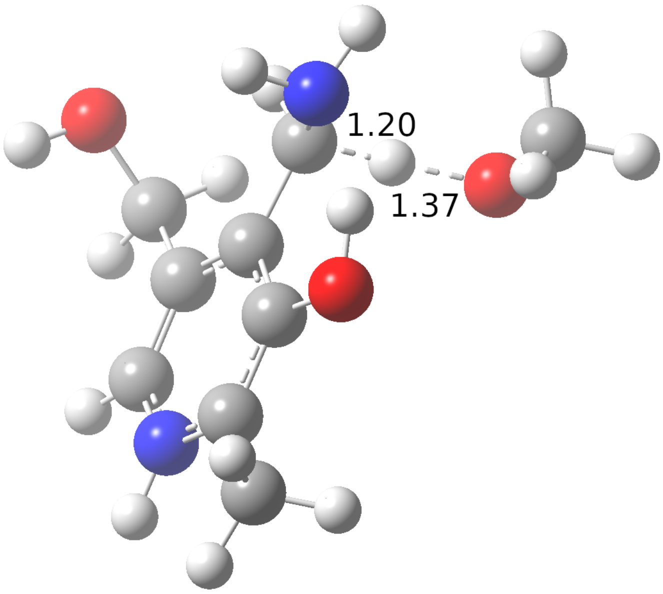

TS-$H_2PM(0)(-H,C8)(OCH_3)^{+\bullet}$

```
Charge=1, Multiplicity=2
C   -0.296674   -1.062314    0.198135
C   -0.191592    0.309275   -0.001346
C   -1.339983    1.004187   -0.405778
C   -2.504687    0.276947   -0.573555
N   -2.600080   -1.043086   -0.378298
H   -3.411226    0.784868   -0.877274
C    1.115555    0.981719    0.165998
H    1.889150    0.481622   -0.662353
H    1.123435    2.040563   -0.054814
C   -1.349788    2.496225   -0.600561
H   -0.577086    2.806219   -1.300607
H   -2.316584    2.797201   -1.002751
O    0.831228   -1.729080    0.566469
O   -1.078604    3.189036    0.622452
H   -1.789736    2.990679    1.240158
N    1.743525    0.758291    1.489531
H    2.671493    1.185191    1.524561
H    1.173834    1.178078    2.229574
C   -1.522273   -1.716391    0.008806
C   -1.621764   -3.192512    0.233758
H   -0.962589   -3.735250   -0.447886
H   -1.335279   -3.452322    1.255525
H   -2.643966   -3.518223    0.063779
H    0.657899   -2.667807    0.710379
H    1.836344   -0.243584    1.682286
O    2.784595   -0.062858   -1.398329
C    3.860353   -0.451572   -0.572667
H    3.560665   -1.201712    0.161035
H    4.590934   -0.907873   -1.249395
H    4.335967    0.405737   -0.092324
```

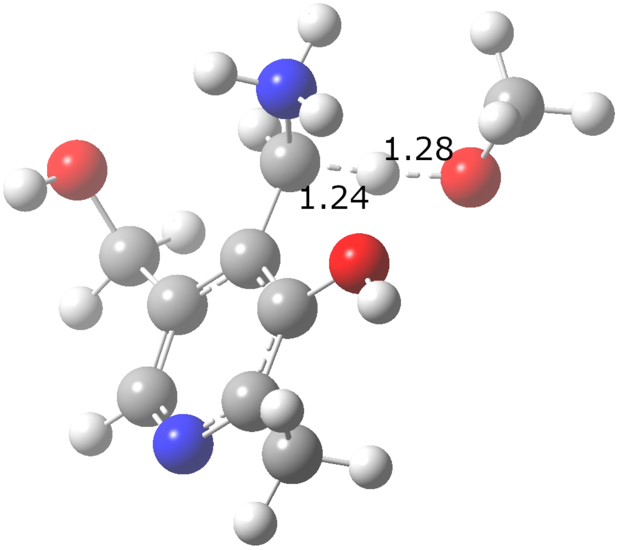

TS-$H_2PM(\pm)(-H,C9)(OCH_3)^{+\bullet}$

Charge=1, Multiplicity=2

C	1.522139	0.504886	-0.282322
C	0.140346	0.475280	0.027986
C	-0.440498	-0.633896	0.649372
C	0.336363	-1.742052	0.904839
N	1.638601	-1.712396	0.565591
H	-0.026731	-2.642868	1.371730
C	-0.648076	1.678889	-0.391685
H	-0.526050	1.840545	-1.460975
H	-1.700098	1.611366	-0.149799
C	-1.878679	-0.669273	1.042135
H	-2.524249	-0.555893	0.058625
H	-2.166040	-1.636375	1.452984
O	2.088130	1.529547	-0.808587
O	-2.182911	0.393922	1.909657
H	-3.085269	0.280310	2.228180
N	-0.093837	2.894660	0.281266
H	-0.548658	3.740251	-0.062380
H	-0.221014	2.841241	1.292105
C	2.264858	-0.675856	0.007073
C	3.708919	-0.773055	-0.319962
H	3.852340	-0.618479	-1.390410
H	4.255741	0.016157	0.197589
H	4.115841	-1.741836	-0.036965
H	0.910224	2.933735	0.063158
H	2.192172	-2.541708	0.760331
O	-3.130794	-0.536000	-1.222904
C	-2.242388	-1.256937	-2.040837
H	-2.036862	-2.250420	-1.635163
H	-2.763379	-1.387791	-2.996964
H	-1.314611	-0.711747	-2.224965

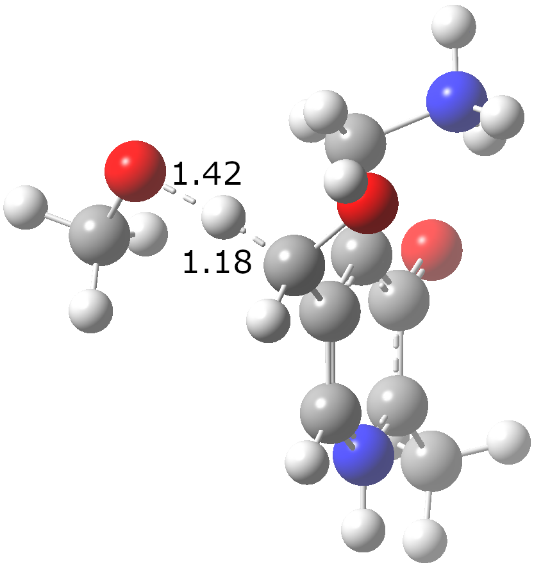

TS-H_2PM(+)(-H,C9)(OCH_3)$^{+\bullet}$

Charge=1, Multiplicity=2

C	1.501172	0.458684	-0.155472
C	0.136342	0.467178	0.146635
C	-0.467799	-0.725287	0.571195
C	0.289207	-1.872793	0.633433
N	1.589740	-1.829953	0.308750
H	-0.102889	-2.830091	0.938528
C	-0.639094	1.741433	-0.069644
H	-0.788832	1.841529	-1.147224
H	-1.614928	1.685473	0.401398
C	-1.910122	-0.810897	0.947907
H	-2.552684	-0.459609	0.016642
H	-2.221271	-1.840898	1.117746
O	2.144418	1.570372	-0.558948
O	-2.192733	0.021350	2.042293
H	-3.092823	-0.157404	2.336905
N	0.146152	2.901594	0.384843
H	-0.281651	3.751343	0.038832
H	0.137438	2.945034	1.397443
C	2.239313	-0.721606	-0.069750
C	3.686728	-0.782167	-0.393852
H	3.842500	-0.501889	-1.435973
H	4.227997	-0.068092	0.226810
H	4.080837	-1.781912	-0.226314
H	2.123723	-2.693063	0.365903
H	1.544975	2.342741	-0.270835
O	-3.189905	-0.196528	-1.210920
C	-2.348319	-0.775108	-2.178673
H	-1.407049	-0.232749	-2.285735
H	-2.167239	-1.833184	-1.974540
H	-2.898167	-0.706894	-3.124819

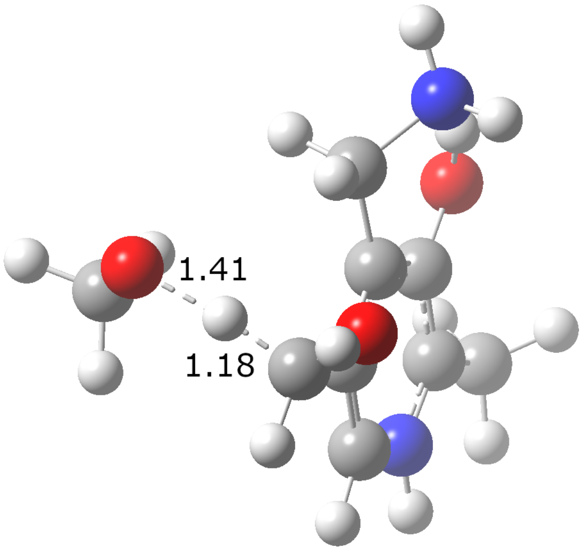

TS-H_2PM(0)(-H,C9)$(OCH_3)^{+\bullet}$

Charge=1, Multiplicity=2

C	1.740453	0.534207	-0.269902
C	0.376110	0.385641	-0.069067
C	-0.090762	-0.875807	0.318036
C	0.825966	-1.908809	0.435195
N	2.133471	-1.764967	0.208271
H	0.490256	-2.894825	0.729104
C	-0.540543	1.556228	-0.282755
H	-0.118135	2.255429	-0.995095
H	-1.517344	1.236470	-0.629203
C	-1.528810	-1.138751	0.592519
H	-2.107492	-1.035990	-0.429797
H	-1.704571	-2.164080	0.914398
O	2.206951	1.770666	-0.604512
O	-2.046711	-0.199076	1.504261
H	-2.945167	-0.451983	1.746490
N	-0.754046	2.299557	0.998299
H	-1.353211	3.110976	0.843010
H	-1.211144	1.675187	1.669413
C	2.600096	-0.568142	-0.135375
C	4.067390	-0.407401	-0.382700
H	4.255082	-0.050057	-1.397966
H	4.504254	0.313759	0.312205
H	4.565381	-1.363894	-0.253309
H	3.159344	1.751269	-0.755251
H	0.131429	2.624436	1.389164
O	-2.995929	-0.760307	-1.519494
C	-4.098382	-0.133924	-0.914579
H	-3.831600	0.826605	-0.468132
H	-4.810237	0.057703	-1.727198
H	-4.586415	-0.782864	-0.184081

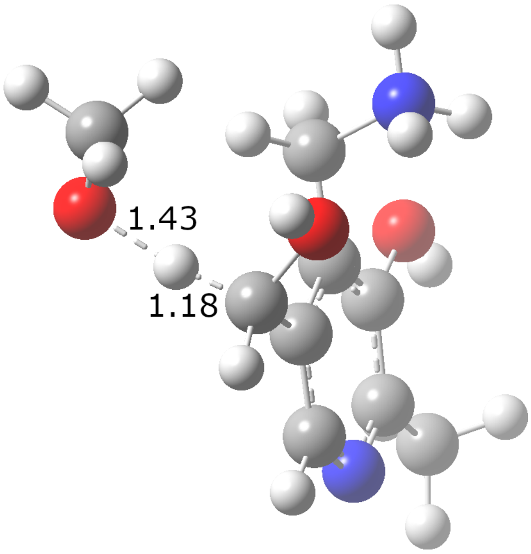

TS-H_2PM(+)(-H,N2)(OCH_3)$^{+\bullet}$

Charge=1, Multiplicity=2

C	-1.359673	-0.927613	-0.137727
C	-0.309986	-0.021703	-0.278677
C	-0.583974	1.351477	-0.240650
C	-1.886408	1.762192	-0.077882
N	-2.861447	0.849450	0.037822
H	-2.186983	2.797349	-0.038484
C	1.094674	-0.546540	-0.441762
H	1.073834	-1.460238	-1.028606
H	1.688460	0.198203	-0.974627
C	0.500901	2.389898	-0.370861
H	1.013389	2.278189	-1.324001
H	0.053316	3.382112	-0.335256
O	-1.075261	-2.249059	-0.164127
O	1.496072	2.248214	0.640474
H	1.098807	2.466962	1.490011
N	1.642904	-0.837689	0.877587
H	2.662709	-1.308813	0.773297
H	1.780621	0.053577	1.352664
C	-2.669540	-0.475611	0.019625
C	-3.833417	-1.388628	0.171829
H	-3.902982	-2.050603	-0.693508
H	-3.706267	-2.003447	1.064583
H	-4.758363	-0.824924	0.258312
H	-3.814480	1.183978	0.153116
H	-1.873239	-2.788786	-0.103609
O	3.930555	-1.346535	0.469494
C	4.276637	-0.524171	-0.619547
H	4.095041	0.531900	-0.403510
H	5.353814	-0.653868	-0.773038
H	3.765912	-0.820686	-1.538023

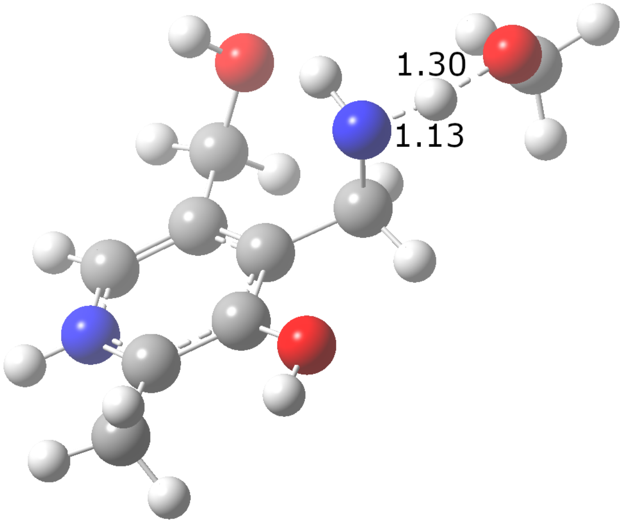

TS-H_2PM(+)(-H,O2)(OCH_3)$^{+\bullet}$

Charge=1, Multiplicity=2

C	-1.825805	0.578519	0.211302
C	-0.453810	0.415044	0.025907
C	0.025914	-0.850450	-0.329673
C	-0.858852	-1.897214	-0.446840
N	-2.165756	-1.683843	-0.243546
H	-0.566664	-2.903194	-0.704012
C	0.440863	1.615937	0.173345
H	0.164985	2.133962	1.094020
H	1.471751	1.295414	0.274395
C	1.492045	-1.121619	-0.559487
H	1.618502	-2.072179	-1.075218
H	1.924892	-0.319589	-1.162245
O	-2.276963	1.815058	0.527228
O	2.094006	-1.151189	0.719154
H	3.281032	-1.091819	0.638127
N	0.327172	2.464752	-1.020900
H	1.026288	3.194764	-0.954042
C	-2.699430	-0.497828	0.081558
C	-4.167456	-0.387970	0.289725
H	-4.372185	-0.043666	1.304994
H	-4.590489	0.335025	-0.409864
H	-4.650469	-1.350095	0.139203
H	-3.235432	1.830444	0.640467
H	-0.572875	2.929590	-1.009304
O	4.306172	-0.769264	0.297182
C	4.367038	0.634148	0.170407
H	5.415566	0.862576	-0.046029
H	3.772006	0.982574	-0.677551
H	4.073181	1.135004	1.092054
H	-2.798758	-2.473407	-0.339851

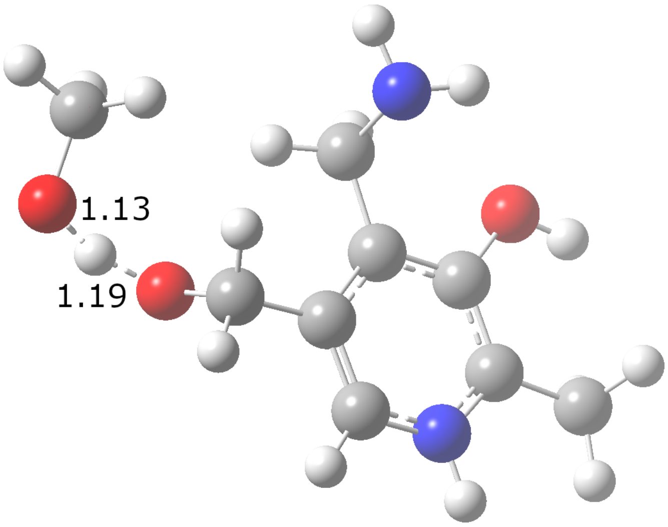

TS-$H_2PM(0)(-H,O2)(OCH_3)^{+\bullet}$

Charge=1, Multiplicity=2

C	1.681716	0.415129	-0.037594
C	0.465813	0.989496	-0.427148
C	-0.504299	0.135273	-0.903265
C	-0.245379	-1.267057	-0.895149
N	0.937281	-1.813450	-0.556002
H	-0.956688	-1.924510	-1.373056
C	0.193851	2.455287	-0.236891
H	1.092932	3.002409	0.018624
H	-0.241243	2.884775	-1.134002
C	-1.926873	0.587644	-1.122450
H	-1.956421	1.536796	-1.659928
H	-2.457660	-0.158026	-1.718264
O	2.635909	1.206548	0.424176
O	-2.498127	0.718251	0.173499
H	-2.062373	-0.393306	0.689663
N	-0.801870	2.625902	0.867176
H	-1.145718	3.584538	0.889241
H	-1.613973	1.934667	0.714587
C	1.883740	-1.013226	-0.124128
C	3.193466	-1.582538	0.290895
H	4.008755	-1.137354	-0.284590
H	3.375884	-1.376161	1.349242
H	3.188300	-2.656178	0.133294
H	3.435126	0.721783	0.677642
H	-0.369554	2.422282	1.767455
O	-1.527649	-1.367570	0.912491
C	-2.429830	-2.449986	0.724951
H	-1.868322	-3.376562	0.611093
H	-3.076636	-2.537484	1.601483
H	-3.054577	-2.292797	-0.157352

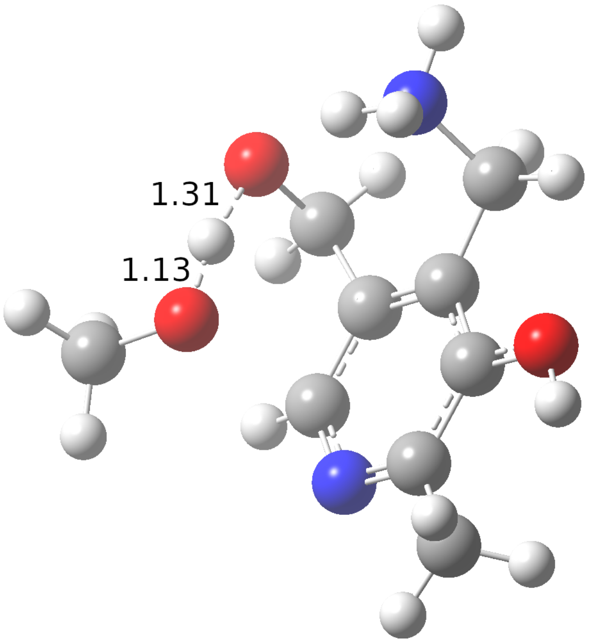

TS-H_2PM(±)(OOH,C2)$^{+\bullet}$

Charge=1, Multiplicity=2

C	-0.933318	0.690547	-0.556703
C	0.480373	0.789564	-0.391358
C	1.274169	-0.346760	-0.331158
C	0.675821	-1.593454	-0.468497
N	-0.650266	-1.683806	-0.656108
H	1.226340	-2.517944	-0.474692
C	1.049635	2.175832	-0.342443
H	2.113348	2.192740	-0.138203
H	0.856957	2.700521	-1.275890
C	2.765181	-0.253263	-0.147125
H	3.196553	0.300539	-0.983567
H	2.978860	0.293131	0.773905
O	-1.679953	1.702669	-0.651194
O	3.315277	-1.561287	-0.083895
H	4.268905	-1.475803	0.005492
C	-1.502587	-0.646813	-0.568555
H	-1.049601	-2.611012	-0.760086
N	0.373361	2.963504	0.734326
H	0.533916	2.544258	1.650360
H	0.716356	3.923796	0.749915
H	-0.634791	2.964300	0.541857
O	-1.792009	-0.575649	1.475674
C	-2.927247	-0.854448	-0.933847
H	-3.216834	-1.888707	-0.759911
H	-3.072588	-0.610149	-1.987780
H	-3.556259	-0.194909	-0.340510
O	-0.701923	-1.101501	2.105867
H	-0.846112	-2.059734	2.118093

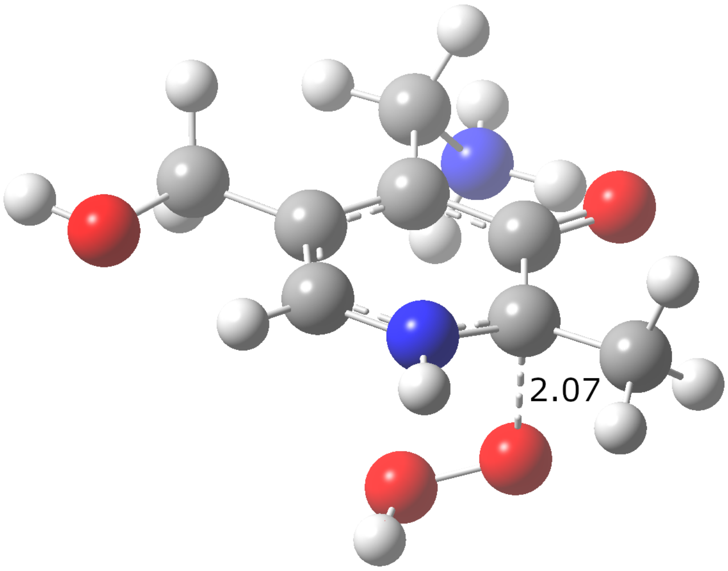

TS-H_2PM(±)(OOH,C4)$^{+\bullet}$

Charge=1, Multiplicity=2

C	-0.509585	1.595809	0.705781
C	-1.020253	0.382280	0.369261
C	1.292370	-0.460160	0.124793
C	1.748071	0.858286	0.458624
N	0.843643	1.791761	0.720335
H	-1.107364	2.447178	0.981941
C	3.193951	1.165185	0.561893
H	3.695836	0.881031	-0.363287
H	3.356410	2.222466	0.758813
H	3.635035	0.575505	1.367164
C	-2.501859	0.151229	0.320420
H	-2.786692	-0.088468	-0.707920
H	-2.761913	-0.699400	0.955294
O	-3.177900	1.319584	0.763010
H	-4.123110	1.150844	0.710021
C	-0.130401	-0.632522	-0.073343
C	-0.579519	-2.050961	-0.279839
H	-1.639181	-2.128923	-0.492079
H	-0.013792	-2.506056	-1.086208
O	2.107375	-1.410862	-0.023521
O	-0.121773	-0.333158	-2.059077
H	1.179575	2.716843	0.969240
N	-0.295017	-2.854548	0.948791
H	-0.575831	-3.826545	0.813787
H	0.708722	-2.833776	1.146442
H	-0.796982	-2.483673	1.756991
O	-0.323870	0.995815	-2.339606
H	0.561600	1.386581	-2.346072

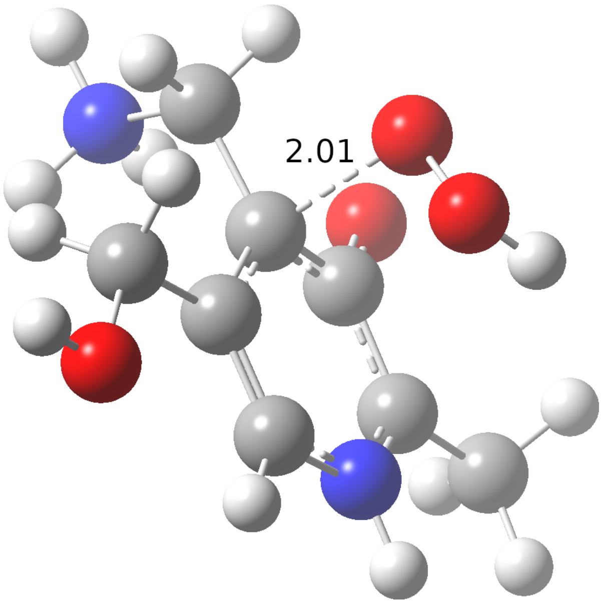

TS-H_2PM(±)(OOH,C6)$^{+\bullet}$

Charge=1, Multiplicity=2

C	-0.380987	-1.527815	-0.830498
C	0.653365	-0.569176	-0.833348
C	-0.976943	1.137983	-0.189166
C	-2.002482	0.141841	-0.338735
N	-1.657895	-1.089513	-0.711295
C	-3.428801	0.476343	-0.117175
H	-3.714942	1.308373	-0.761643
H	-4.068675	-0.379802	-0.318839
H	-3.570329	0.801170	0.914760
C	2.058621	-1.047354	-1.062209
H	2.059128	-2.124070	-1.225311
H	2.484147	-0.556646	-1.939284
O	2.829598	-0.720832	0.101921
H	3.754113	-0.913035	-0.086424
C	0.364649	0.735152	-0.521588
C	1.423055	1.793902	-0.462310
H	2.259649	1.572028	-1.115699
H	1.007310	2.763842	-0.711930
O	-1.269929	2.302081	0.195601
H	-2.399553	-1.774868	-0.817606
N	1.966531	1.885166	0.929349
H	2.669731	2.621152	0.996467
H	1.221805	2.087502	1.597855
H	2.400788	0.986093	1.160976
O	-0.039469	-2.240168	1.061994
H	-0.251758	-2.516395	-1.238543
O	-0.388512	-1.209479	1.897712
H	-1.328230	-1.342991	2.091185

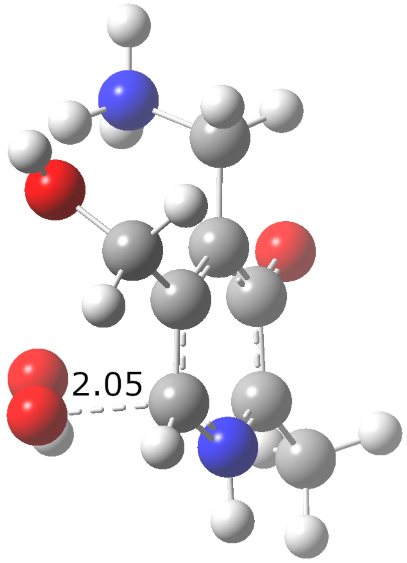

TS-H_2PM(±)($OOCH_3$,C2)$^{+\bullet}$

Charge=1, Multiplicity=2

C	-0.332244	1.279379	-0.540923
C	0.968451	0.752889	-0.286132
C	1.237014	-0.598321	-0.453751
C	0.228416	-1.431385	-0.921927
N	-0.983518	-0.925963	-1.202645
H	0.370734	-2.479092	-1.122683
C	2.014214	1.741732	0.133433
H	2.962505	1.280204	0.380705
H	2.170625	2.487177	-0.643212
C	2.600496	-1.168226	-0.167464
H	3.334011	-0.692132	-0.821364
H	2.869404	-0.950046	0.868110
O	-0.606307	2.502753	-0.421844
O	2.580818	-2.571469	-0.386767
H	3.457377	-2.911703	-0.186268
C	-1.374843	0.324436	-0.895040
H	-1.690257	-1.561257	-1.558149
N	1.547480	2.482537	1.347006
H	1.416516	1.848160	2.135340
H	2.221446	3.199328	1.616055
H	0.649892	2.927995	1.126631
O	-1.869894	0.123942	1.078513
C	-2.696757	0.800184	-1.379349
H	-3.392418	-0.033294	-1.459710
H	-2.578223	1.270033	-2.357552
H	-3.093707	1.541378	-0.689982
O	-1.225308	-0.967601	1.575735
C	-2.105176	-2.099175	1.555078
H	-2.477983	-2.265896	0.546091
H	-1.505837	-2.943023	1.887469
H	-2.933716	-1.923934	2.239348

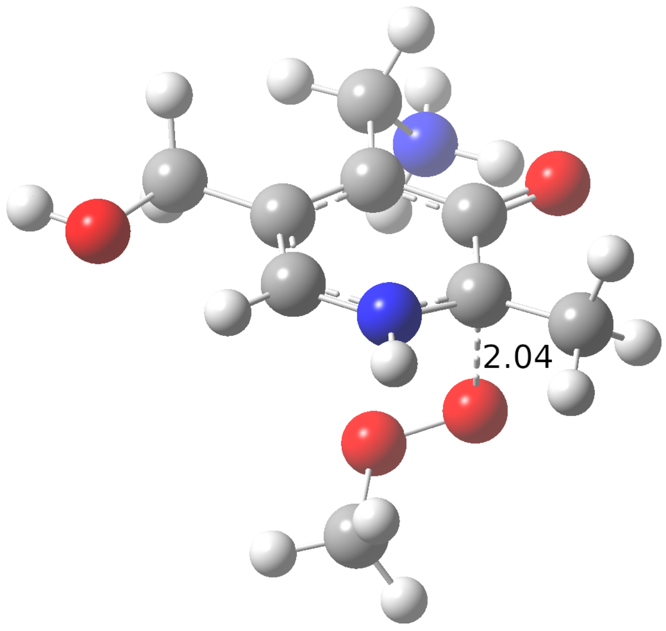

TS-H_2PM(±)($OOCH_3$,C6)$^{+\bullet}$

Charge=1, Multiplicity=2

C	-0.302833	1.243874	-0.992245
C	-1.159682	0.174569	-0.632950
C	0.807521	-1.264430	-0.433181
C	1.606789	-0.173429	-0.901130
N	1.000273	0.966974	-1.232160
C	3.075463	-0.298624	-1.046763
H	3.524134	-0.454090	-0.063344
H	3.316763	-1.168485	-1.657938
H	3.501384	0.594957	-1.497298
C	-2.632156	0.470315	-0.458839
H	-3.203657	-0.042655	-1.230854
H	-2.959736	0.097067	0.512615
O	-2.930941	1.851806	-0.577223
H	-2.547356	2.307050	0.180908
C	-0.613428	-1.051255	-0.362550
C	-1.432731	-2.240888	0.039443
H	-2.496093	-2.037442	0.070792
H	-1.247451	-3.071775	-0.636908
O	1.340105	-2.364326	-0.102783
H	1.580842	1.724956	-1.578053
N	-1.016756	-2.701622	1.400712
H	-1.537529	-3.534588	1.675104
H	-0.015505	-2.921475	1.373980
H	-1.177546	-1.974270	2.098358
O	-0.234786	2.062701	0.861820
H	-0.677108	2.159215	-1.415839
O	0.595586	1.226669	1.564317
C	1.874048	1.852654	1.732959
H	1.767125	2.726840	2.373243
H	2.503794	1.105733	2.210796
H	2.285663	2.139977	0.767053

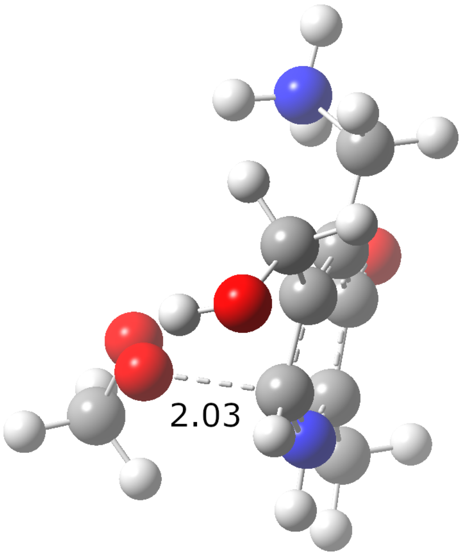

TS-H_2PM(±)(OCH_3,C2)$^{+\bullet}$

Charge=1, Multiplicity=2

C	-0.973219	0.517224	-0.666446
C	0.425418	0.734070	-0.485147
C	1.308829	-0.327689	-0.347216
C	0.810095	-1.621940	-0.388648
N	-0.507252	-1.819915	-0.546488
H	1.425172	-2.502802	-0.316640
C	0.878943	2.163850	-0.487870
H	1.927769	2.280156	-0.243624
H	0.684003	2.624192	-1.454303
C	2.793108	-0.104769	-0.156017
H	3.188516	0.433623	-1.015606
H	2.952299	0.509479	0.731851
O	-1.791051	1.469334	-0.848399
O	3.527695	-1.313310	-0.061228
H	3.370141	-1.708913	0.802177
C	-1.429180	-0.847617	-0.606786
H	-0.840963	-2.778824	-0.574190
N	0.089843	2.936641	0.520351
H	0.246476	2.575075	1.461635
H	0.342886	3.924262	0.501699
H	-0.905282	2.828867	0.284897
O	-1.818443	-0.519547	1.577724
C	-0.669150	-0.551736	2.362043
H	-0.129434	0.399142	2.372064
H	-0.977938	-0.805990	3.385542
H	0.005864	-1.361877	2.050392
C	-2.836340	-1.205267	-0.915064
H	-3.044355	-2.232142	-0.619629
H	-3.011074	-1.100214	-1.987961
H	-3.508841	-0.529680	-0.393221

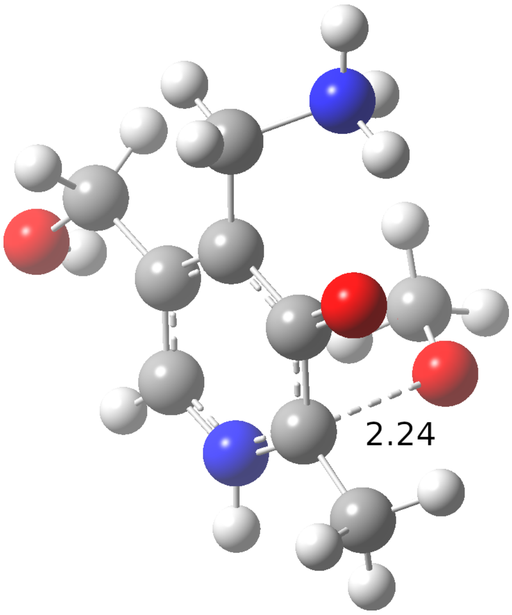

TS-$H_2PM(+)(OCH_3,C2)^{+\bullet}$

Charge=1, Multiplicity=2

C	-0.886944	0.668217	-0.600248
C	0.504553	0.711622	-0.523633
C	1.197699	-0.494868	-0.373000
C	0.493696	-1.689649	-0.360739
N	-0.833878	-1.683730	-0.484858
H	0.968745	-2.653086	-0.281396
C	1.238894	2.028642	-0.556994
H	2.218850	1.877430	-1.002044
H	0.714320	2.729726	-1.211472
C	2.694556	-0.513666	-0.261219
H	3.124689	-0.192903	-1.213196
H	3.004109	0.190377	0.511943
O	-1.674269	1.740857	-0.732694
O	3.120556	-1.829899	0.057003
H	4.081976	-1.830775	0.083216
C	-1.593573	-0.563000	-0.506353
H	-1.321309	-2.575235	-0.496536
N	1.413790	2.530662	0.812521
H	1.890916	3.423145	0.766112
H	0.501332	2.710512	1.216558
O	-2.007293	-0.321342	1.453809
C	-0.897784	-0.374205	2.302678
H	-0.311245	0.546118	2.289687
H	-1.300012	-0.527433	3.312714
H	-0.261330	-1.242969	2.094934
C	-3.018971	-0.690395	-0.918769
H	-3.409666	-1.654908	-0.601096
H	-3.080273	-0.616375	-2.005514
H	-3.607692	0.106244	-0.475697
H	-1.177499	2.568494	-0.717137

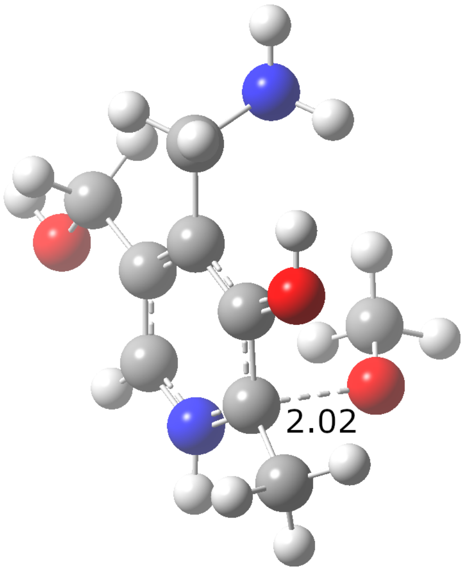

TS-H_2PM(0)(OCH_3,C2)$^{+\bullet}$

Charge=1, Multiplicity=2

C	-0.917253	0.584761	-0.629224
C	0.470254	0.652539	-0.549131
C	1.163184	-0.548590	-0.370614
C	0.430406	-1.744935	-0.322937
N	-0.883714	-1.816621	-0.428740
H	0.958683	-2.681497	-0.225490
C	1.201257	1.964997	-0.647800
H	2.212676	1.830972	-1.014651
H	0.705930	2.674014	-1.305172
C	2.663297	-0.569168	-0.269802
H	3.092357	-0.238357	-1.219285
H	2.991765	0.121253	0.510226
O	-1.727089	1.644547	-0.810379
O	3.106065	-1.884876	0.034076
H	4.066929	-1.868210	0.069770
C	-1.590425	-0.668559	-0.493833
N	1.308080	2.617800	0.699733
H	1.798511	3.510980	0.629082
H	0.381071	2.788908	1.094583
O	-2.000698	-0.327448	1.475912
C	-0.869559	-0.299586	2.296289
H	-0.350748	0.663044	2.271898
H	-1.207244	-0.494770	3.321562
H	-0.167277	-1.105524	2.046704
C	-3.031609	-0.767180	-0.882551
H	-3.432880	-1.713105	-0.527992
H	-3.113049	-0.737648	-1.971296
H	-3.611352	0.053636	-0.470992
H	-1.255636	2.484199	-0.840056
H	1.816153	2.020389	1.354906

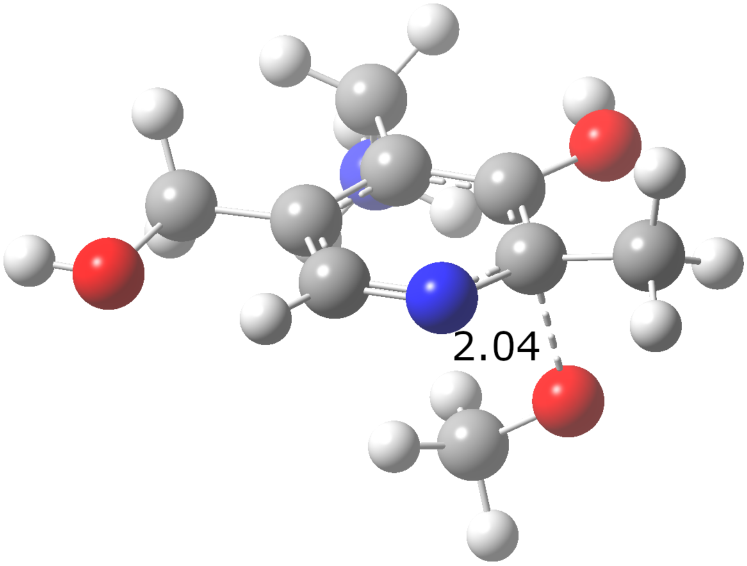

TS-H_2PM(0)(OCH_3,C3)$^{+\bullet}$

Charge=1, Multiplicity=2

C	0.550650	2.056306	-0.424203
C	1.277143	0.870982	-0.265684
C	-0.837883	-0.248904	-0.313387
C	-1.469935	1.004341	-0.620341
N	-0.773507	2.124114	-0.631212
H	1.081949	2.998809	-0.426703
C	-2.937004	1.033676	-0.868777
H	-3.187559	0.455609	-1.761984
H	-3.475744	0.592148	-0.025608
H	-3.267492	2.058946	-1.005072
C	2.767477	0.931190	-0.093982
H	3.258801	0.237052	-0.777501
H	3.115335	1.940282	-0.312921
O	3.072299	0.584132	1.262661
H	4.030016	0.585126	1.359886
C	0.580422	-0.318917	-0.261543
C	1.219555	-1.662250	-0.092174
H	2.281511	-1.585303	0.103714
H	0.743391	-2.210538	0.717768
O	-1.472137	-1.423158	-0.526466
O	-1.089939	0.076205	1.651174
C	-1.939805	-0.921688	2.152017
H	-2.072421	-0.706685	3.219896
H	-2.932346	-0.900471	1.690176
H	-1.511480	-1.923529	2.061961
N	1.055185	-2.486798	-1.332289
H	1.489114	-2.026408	-2.133875
H	1.495266	-3.399883	-1.211309
H	-2.430015	-1.313180	-0.585933
H	0.064436	-2.629037	-1.538489

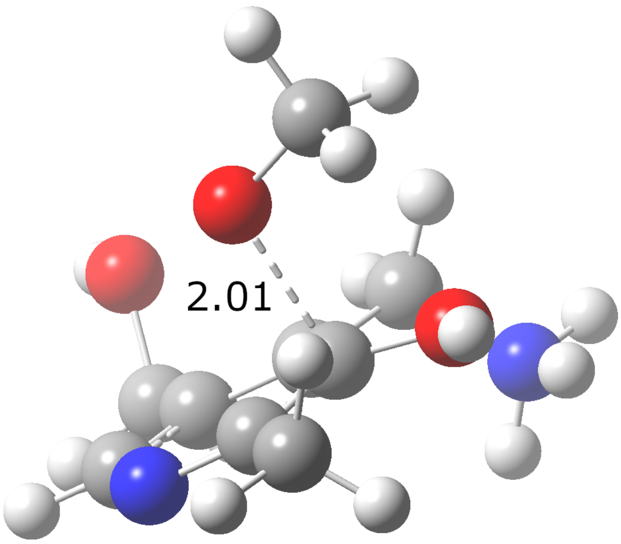

TS-H_2PM(+)(OCH_3,C4)$^{+\bullet}$

Charge=1, Multiplicity=2

C	0.962284	-1.655821	0.292968
C	1.211016	-0.320347	0.364869
C	-1.202091	0.051539	0.419593
C	-1.399293	-1.336966	0.338893
N	-0.320017	-2.111034	0.278109
H	1.730175	-2.410648	0.261776
C	-2.756700	-1.935077	0.331714
H	-3.300337	-1.624673	1.223901
H	-3.307940	-1.572858	-0.537140
H	-2.698584	-3.019980	0.300970
C	2.621528	0.208344	0.383444
H	2.756058	0.889094	-0.460049
H	2.783923	0.773971	1.300453
O	3.596702	-0.818962	0.357051
H	3.630334	-1.191513	-0.529971
C	0.114118	0.592551	0.350935
C	0.334749	2.031927	0.749124
H	0.453267	2.052712	1.833096
H	1.251372	2.407014	0.300127
O	-2.266707	0.827716	0.504331
O	0.043194	1.036447	-1.605939
C	-0.165066	-0.056425	-2.461098
H	-0.012567	0.333895	-3.474339
H	0.573349	-0.848842	-2.306712
H	-1.180403	-0.454413	-2.394769
H	-0.464994	-3.115227	0.224655
N	-0.827407	2.863414	0.392395
H	-0.861284	3.684692	0.982639
H	-0.751057	3.163686	-0.571832
H	-1.878114	1.826469	0.487467

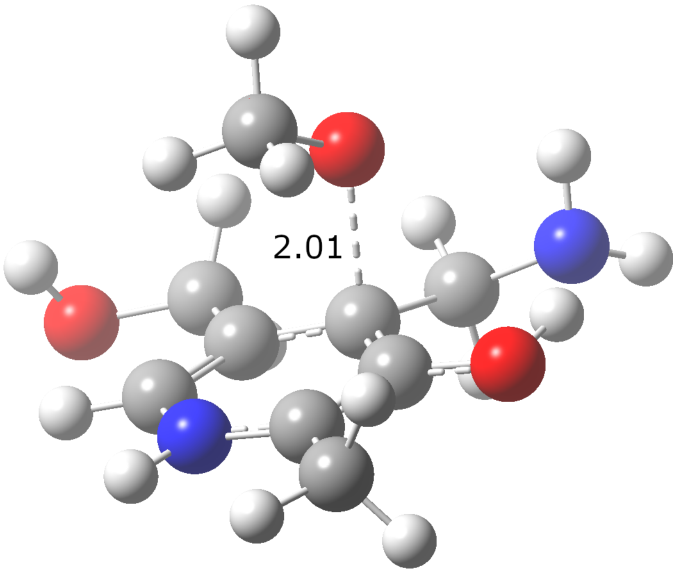

TS-H_2PM(0)(OCH_3,C4)$^{+\bullet}$

Charge=1, Multiplicity=2

C	0.453561	-1.607908	0.774866
C	0.996181	-0.425800	0.355464
C	-1.272678	0.369170	0.107507
C	-1.726178	-0.889133	0.550985
N	-0.877998	-1.845018	0.878386
H	1.094508	-2.429822	1.057538
C	-3.197554	-1.131813	0.667765
H	-3.651938	-0.421853	1.360023
H	-3.685610	-1.003384	-0.299430
H	-3.370806	-2.142346	1.026888
C	2.485970	-0.230534	0.224695
H	2.717011	0.016196	-0.812878
H	2.813231	0.605943	0.847797
O	3.235702	-1.392144	0.540205
H	3.197604	-1.537993	1.490957
C	0.112698	0.612328	-0.080634
C	0.621622	2.028210	-0.202340
H	1.642547	2.047236	-0.564091
H	0.018800	2.627625	-0.875633
O	-2.228896	1.276327	-0.165638
O	0.115047	0.428353	-2.025069
C	0.153279	-0.918178	-2.432478
H	0.101958	-0.896020	-3.526471
H	1.079674	-1.417619	-2.140693
H	-0.710805	-1.482226	-2.071103
N	0.605030	2.702401	1.135000
H	-0.337612	2.723736	1.529205
H	1.214327	2.215478	1.795528
H	-1.883225	2.089752	-0.551448
H	0.934885	3.665266	1.049134

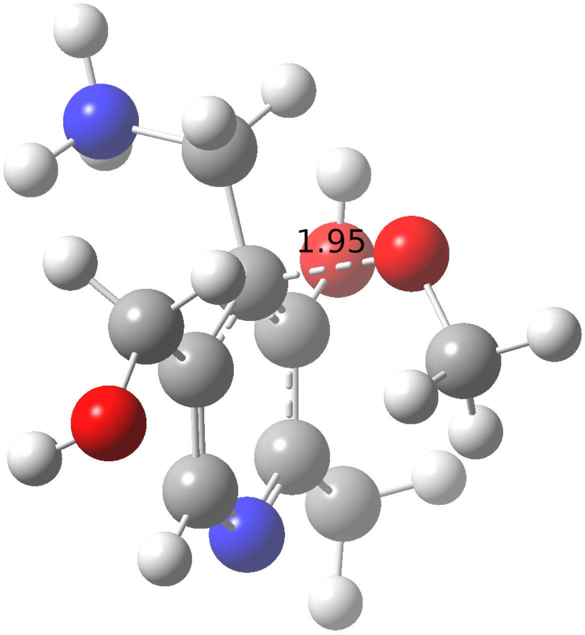

TS-H_2PM(±)(OCH_3,C4)$^{+•}$

Charge=1, Multiplicity=2

C	0.518441	1.557154	0.804105
C	1.010675	0.343254	0.421015
C	-1.305897	-0.458473	0.117591
C	-1.743305	0.854946	0.500650
N	-0.828882	1.759723	0.817457
H	1.124608	2.391190	1.111361
C	-3.185674	1.183771	0.588104
H	-3.664804	0.981777	-0.370572
H	-3.661057	0.543011	1.332232
H	-3.336062	2.226293	0.859322
C	2.489581	0.090429	0.373699
H	2.730237	-0.774397	0.997225
H	2.775150	-0.140165	-0.656518
O	3.182376	1.240999	0.835271
H	4.124785	1.054590	0.791217
C	0.109051	-0.648761	-0.024321
O	-2.139866	-1.384319	-0.093095
H	-1.157907	2.678402	1.100477
C	0.542424	-2.053164	-0.322273
H	1.599395	-2.135019	-0.545517
H	-0.036262	-2.455038	-1.145588
N	0.256390	-2.923026	0.864346
H	-0.743996	-2.891682	1.075966
H	0.513549	-3.891594	0.670244
O	0.079302	-0.240336	-2.152717
C	0.351952	1.104475	-2.421541
H	0.353478	1.200955	-3.515920
H	-0.423604	1.774331	-2.038041
H	1.336235	1.417298	-2.061551
H	0.775718	-2.609367	1.685198

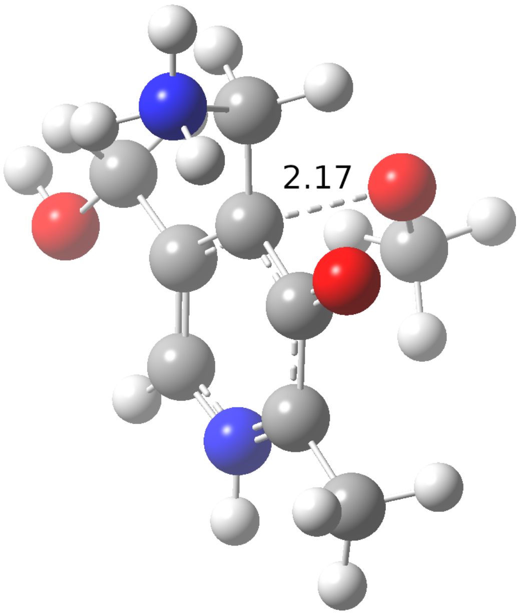

TS-H_2PM(±)(OCH_3,C6)$^{+\bullet}$

Charge=1, Multiplicity=2			
C	-0.366634	-1.478343	-0.955664
C	0.621385	-0.494619	-0.899562
C	-1.059466	1.123272	-0.151221
C	-2.042377	0.084988	-0.320221
N	-1.651722	-1.113261	-0.740305
C	-3.474683	0.337380	-0.035033
H	-3.813863	1.206189	-0.599610
H	-4.082906	-0.527269	-0.290454
H	-3.599783	0.567942	1.024429
C	2.041706	-0.891578	-1.184412
H	2.087446	-1.954976	-1.414233
H	2.424614	-0.328525	-2.037109
O	2.821845	-0.601228	-0.016730
H	3.750943	-0.723144	-0.237946
C	0.283948	0.790597	-0.516435
C	1.302528	1.885210	-0.420685
H	2.132117	1.736416	-1.103157
H	0.841795	2.849314	-0.606555
O	-1.402227	2.258375	0.287198
H	-2.364639	-1.829274	-0.847388
N	1.875935	1.923483	0.962194
H	2.525118	2.703039	1.069062
H	1.135628	2.019148	1.658734
H	2.374720	1.041754	1.121623
O	0.134248	-2.403836	1.011629
C	-0.093200	-1.427545	1.980203
H	-0.041558	-1.938574	2.952466
H	0.658601	-0.634182	1.972039
H	-1.102461	-1.001788	1.911206
H	-0.205434	-2.460282	-1.364936

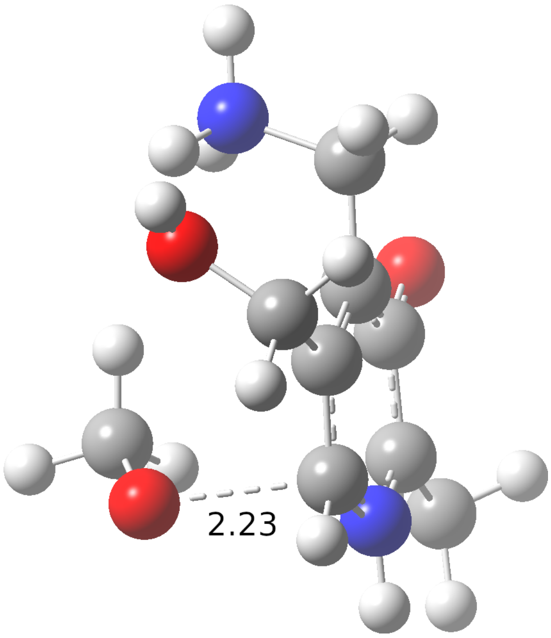

TS-H_2PM(+)(OCH_3,C6)$^{+\bullet}$

Charge=1, Multiplicity=2

C	0.582608	-1.332038	-0.692927
C	0.863902	0.041583	-0.669374
C	-1.458518	0.463630	-0.263701
C	-1.732421	-0.915038	-0.346016
N	-0.712699	-1.728213	-0.604271
C	-3.101263	-1.461135	-0.187270
H	-3.464861	-1.240625	0.817140
H	-3.771585	-0.976116	-0.896730
H	-3.108704	-2.535962	-0.350020
C	2.299846	0.485555	-0.777286
H	2.439813	1.046812	-1.700611
H	2.520415	1.150876	0.060242
O	3.206861	-0.601185	-0.805522
H	3.080406	-1.098593	0.014152
C	-0.162344	0.954607	-0.446543
C	0.106458	2.436180	-0.365158
H	0.994285	2.670084	-0.945639
H	-0.717869	2.976796	-0.839531
O	-2.530158	1.230445	-0.009209
H	-0.909813	-2.721357	-0.691132
N	0.338097	2.834853	1.029987
H	0.544051	3.826684	1.044176
H	-0.519966	2.714168	1.556362
O	1.378444	-1.731306	1.121621
C	0.697731	-1.039421	2.133602
H	0.982059	-1.528671	3.073292
H	0.975268	0.014605	2.183639
H	-0.389490	-1.147412	2.043140
H	1.268372	-2.057726	-1.093061
H	-2.317333	2.172789	-0.022524

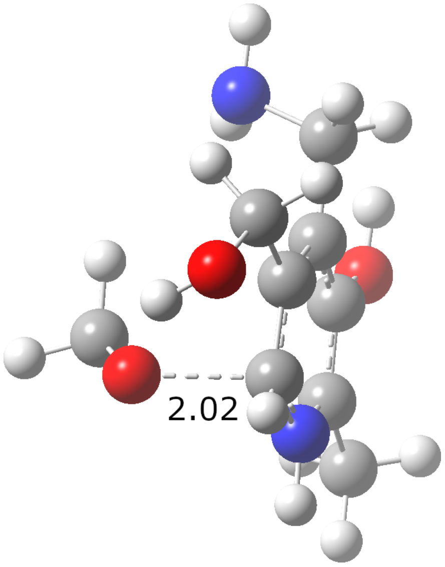

TS-H_2PM(0)(OCH_3,C6)$^{+\bullet}$

Charge=1, Multiplicity=2

C	0.332861	1.531052	-0.878527
C	-0.630441	0.502768	-0.860639
C	1.112675	-0.969123	-0.189120
C	2.039814	0.101818	-0.331739
N	1.658044	1.299675	-0.713089
C	3.486043	-0.157721	-0.059445
H	3.624376	-0.483660	0.972537
H	3.863850	-0.952182	-0.704120
H	4.056016	0.751171	-0.229006
C	-2.057394	0.845552	-1.163480
H	-2.132824	1.907268	-1.392622
H	-2.409998	0.273446	-2.024017
O	-2.856022	0.531394	-0.012444
H	-3.783833	0.611458	-0.257694
C	-0.236304	-0.779372	-0.490848
C	-1.230322	-1.904887	-0.411397
H	-2.025702	-1.773590	-1.137546
H	-0.775304	-2.875676	-0.584125
O	1.646840	-2.137930	0.221939
N	-1.874679	-1.939196	0.938643
H	-2.520267	-2.726023	1.010706
H	-1.176583	-2.020484	1.678879
O	-0.237971	2.326214	0.899587
C	0.061355	1.448483	1.949146
H	-0.029919	2.029988	2.875121
H	-0.627218	0.600875	2.005722
H	1.094976	1.086891	1.904676
H	0.071598	2.476571	-1.328567
H	0.988914	-2.836988	0.314057
H	-2.394132	-1.061257	1.060762

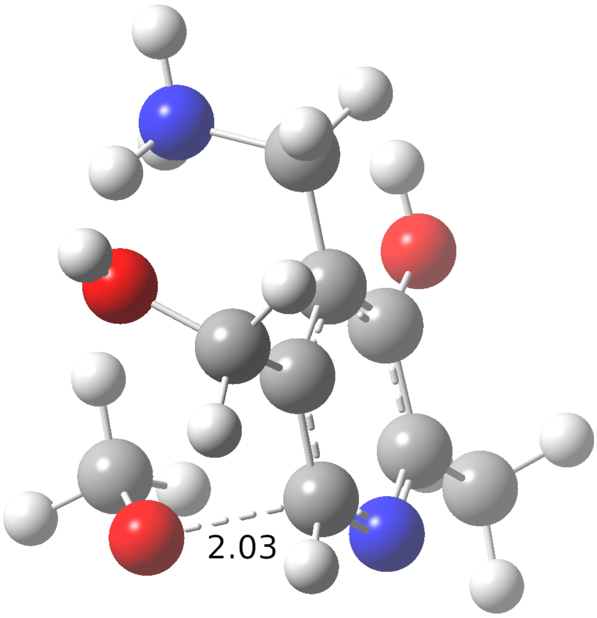

Cartesian coordinates and structures of the optimized geometries in pentyl ethanoate of the located transition states. In the pictures, distances are given in Å.

TS-HPM(0)(-H,C8)(OOH)•

```
Charge=0, Multiplicity=2
C   -1.482235   -0.665390   -0.088443
C   -0.190359   -0.221964   -0.396994
C    0.017167    1.166516   -0.426960
C   -1.060796    2.000063   -0.167743
N   -2.290584    1.570511    0.116949
H   -0.920573    3.075088   -0.196970
C    0.847980   -1.245871   -0.759438
H    0.813362   -1.515652   -1.815771
H    2.044839   -0.792899   -0.656501
C    1.357005    1.814397   -0.651575
H    1.979897    1.259451   -1.343857
H    1.209084    2.818694   -1.049409
O   -1.828551   -1.970928   -0.055301
O    2.111812    1.897793    0.572318
H    1.605118    2.423415    1.199755
N    0.809036   -2.402934    0.070030
H    1.413525   -3.142469   -0.262499
H    1.034677   -2.190372    1.036280
C   -2.508016    0.263095    0.162405
C   -3.886310   -0.232477    0.474010
H   -4.284781   -0.815906   -0.357509
H   -3.873640   -0.886539    1.346851
H   -4.537906    0.616691    0.664048
H   -0.999276   -2.494831   -0.019221
O    3.237464   -0.631433   -0.317269
O    3.156627   -0.566053    1.060531
H    2.901074    0.367038    1.192152
```

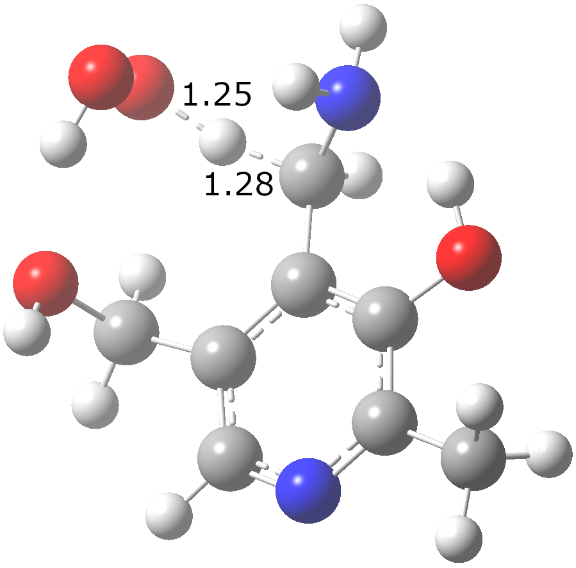

TS-HPM(0)(-H,C9)(OOH)•

Charge=0, Multiplicity=2

C	1.715424	0.472082	-0.147855
C	0.335809	0.365833	0.019047
C	-0.189949	-0.915571	0.234938
C	0.680958	-2.000150	0.216248
N	1.994992	-1.891184	0.028199
H	0.294929	-3.000823	0.370797
C	-0.516113	1.605781	-0.115566
H	-0.538909	1.881664	-1.172704
H	-1.534063	1.402987	0.205310
C	-1.631525	-1.182914	0.451269
H	-2.254359	-0.855314	-0.586424
H	-1.857802	-2.247475	0.526328
O	2.319606	1.662881	-0.352273
O	-2.187295	-0.435040	1.481287
H	-3.121311	-0.662192	1.554304
N	0.097031	2.726218	0.617901
H	-0.375158	3.594219	0.400689
H	0.020910	2.577752	1.617313
C	2.517901	-0.680940	-0.141579
C	3.994907	-0.552280	-0.347400
H	4.457480	-1.532780	-0.265376
H	4.213282	-0.130824	-1.330132
H	4.429287	0.120911	0.392865
H	1.682637	2.352301	-0.027282
O	-3.165159	-0.404848	-1.406725
O	-4.156985	0.049999	-0.563972
H	-3.986894	0.999358	-0.489536

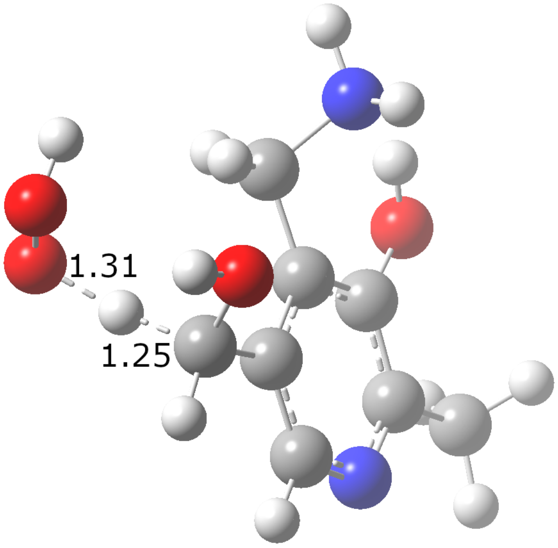

TS-HPM(0)(-H,C7)(OCH_3)•

Charge=0, Multiplicity=2

C	0.291749	0.221862	-0.586564
C	-1.083838	0.359318	-0.390848
C	-1.818903	-0.797889	-0.119319
C	-1.145450	-2.014547	-0.072953
N	0.160843	-2.148463	-0.281679
H	-1.698192	-2.923252	0.138272
C	-1.724129	1.720993	-0.526563
H	-1.785674	1.963872	-1.590252
H	-2.732528	1.705414	-0.120162
C	-3.302978	-0.763787	0.137420
H	-3.828446	-0.244279	-0.661843
H	-3.683085	-1.786300	0.176714
O	1.084567	1.282234	-0.843625
O	-3.631030	-0.062582	1.338854
H	-3.183177	-0.500508	2.068052
N	-0.880503	2.749417	0.108572
H	-1.213355	3.675040	-0.128727
H	-0.920773	2.662371	1.117452
C	0.880020	-1.055236	-0.539422
C	2.331747	-1.201671	-0.751550
H	2.745928	-0.518472	-1.488592
H	2.876836	-0.880916	0.269723
H	2.620528	-2.235426	-0.917563
H	0.571219	2.084087	-0.547311
O	3.622462	-0.339809	1.274220
C	4.381158	0.676568	0.688690
H	4.937012	1.142857	1.511552
H	5.110407	0.292376	-0.030184
H	3.756662	1.444050	0.225681

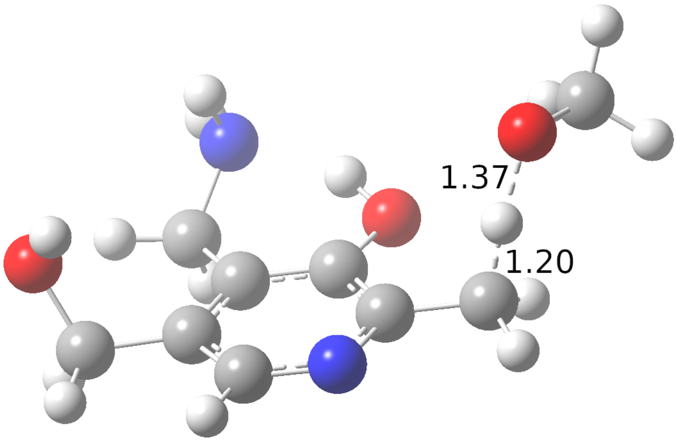

TS-HPM(0)(-H,C8)(OCH_3)•

Charge=0, Multiplicity=2

C	-1.072905	-0.309409	0.464120
C	0.255349	0.105999	0.327290
C	0.487209	1.344548	-0.298350
C	-0.603170	2.084759	-0.716309
N	-1.873425	1.687997	-0.573135
H	-0.449659	3.044753	-1.195937
C	1.379965	-0.776520	0.731917
H	1.572358	-1.511215	-0.195313
H	2.320814	-0.239483	0.831788
C	1.878108	1.883043	-0.508665
H	2.513983	1.147053	-0.998399
H	1.826652	2.761289	-1.154765
O	-1.416461	-1.494056	1.006180
O	2.531728	2.204519	0.721089
H	1.997799	2.861467	1.176406
N	1.063190	-1.651861	1.853751
H	1.745530	-2.395880	1.927472
H	1.084519	-1.132368	2.725709
C	-2.114915	0.519455	0.003069
C	-3.533302	0.064552	0.156026
H	-3.702266	-0.864226	-0.391749
H	-3.765224	-0.135188	1.203047
H	-4.200229	0.833751	-0.225444
H	-0.591744	-1.865440	1.421252
O	1.617943	-2.379595	-1.256493
C	0.474694	-2.115778	-2.010214
H	-0.444332	-2.417440	-1.501899
H	0.416221	-1.069319	-2.325387
H	0.575358	-2.721547	-2.920608

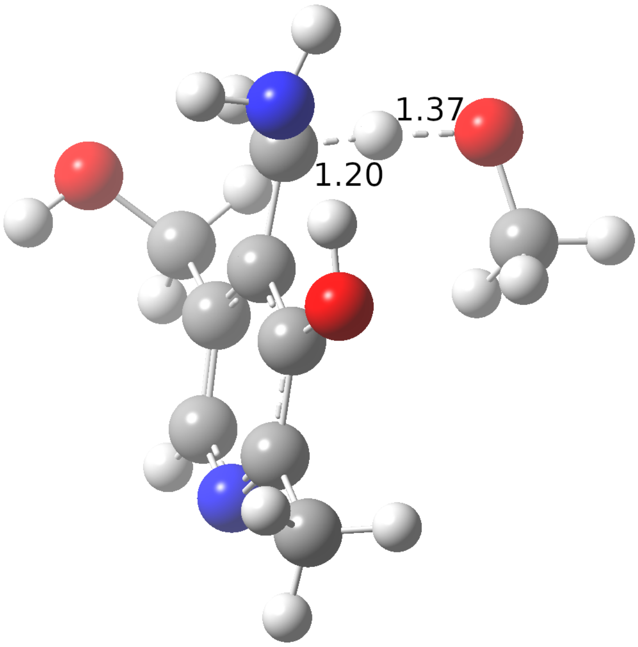

TS-HPM(0)(-H,C9)(OCH_3)•

```
Charge=0, Multiplicity=2
C     1.787285   -0.263322   -0.181709
C     0.703260    0.612605   -0.207477
C    -0.554576    0.094435    0.131225
C    -0.651764   -1.249026    0.464416
N     0.390297   -2.083690    0.475334
H    -1.614891   -1.666836    0.730916
C     0.909331    2.045972   -0.639104
H     1.028670    2.060838   -1.725097
H     0.038898    2.649193   -0.388475
C    -1.774544    0.969277    0.171208
H    -1.945626    1.532009   -0.744637
H    -2.732950    0.254010    0.257557
O     3.038744    0.134606   -0.499692
O    -1.796708    1.871459    1.239194
H    -1.531988    1.404178    2.038791
N     2.150234    2.584179   -0.055833
H     2.381931    3.473919   -0.478161
H     2.034046    2.741615    0.938357
C     1.591524   -1.611497    0.162914
C     2.761498   -2.546328    0.171807
H     3.216723   -2.607571   -0.817955
H     3.532958   -2.192188    0.857074
H     2.428773   -3.535114    0.477913
H     3.042715    1.123867   -0.405530
O    -3.827243   -0.559123    0.185997
C    -3.936231   -0.918406   -1.161536
H    -4.135853   -0.059599   -1.808338
H    -3.060879   -1.467841   -1.520065
H    -4.799297   -1.591697   -1.223317
```

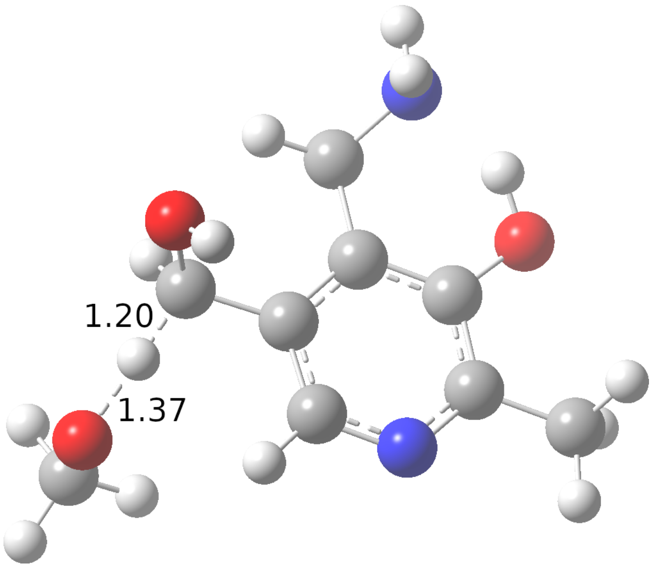

TS-HPM(0)(-H,N2)(OCH_3)•

Charge=0, Multiplicity=2

C	-1.346571	0.452381	0.428065
C	-0.074468	0.067142	0.843646
C	0.327709	-1.238992	0.555259
C	-0.566252	-2.056123	-0.132492
N	-1.777149	-1.676404	-0.528759
H	-0.277855	-3.073293	-0.372213
C	0.792949	1.098723	1.535216
H	0.231315	1.530225	2.367742
H	1.695246	0.649614	1.937723
C	1.690487	-1.789298	0.898624
H	2.041297	-1.392312	1.853134
H	1.614371	-2.870013	1.005566
O	-1.735879	1.731438	0.708165
O	2.643253	-1.552444	-0.128674
H	2.583650	-0.621877	-0.401804
N	1.179681	2.197302	0.661012
H	1.925897	1.891255	-0.097535
H	0.361651	2.585014	0.198204
C	-2.178446	-0.435875	-0.255756
C	-3.549365	-0.012390	-0.696604
H	-4.179438	0.258925	0.154898
H	-3.506926	0.841092	-1.378572
H	-4.022787	-0.840558	-1.217378
H	-2.639163	1.881803	0.410814
O	2.304855	1.068468	-1.082204
C	1.413495	1.012463	-2.165099
H	1.098370	2.008957	-2.484681
H	1.959522	0.549554	-2.995399
H	0.542069	0.387442	-1.949722

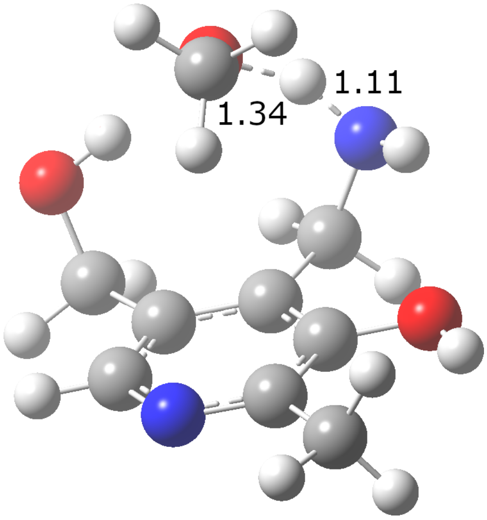

TS-HPM(0)(OCH_3,C2)•

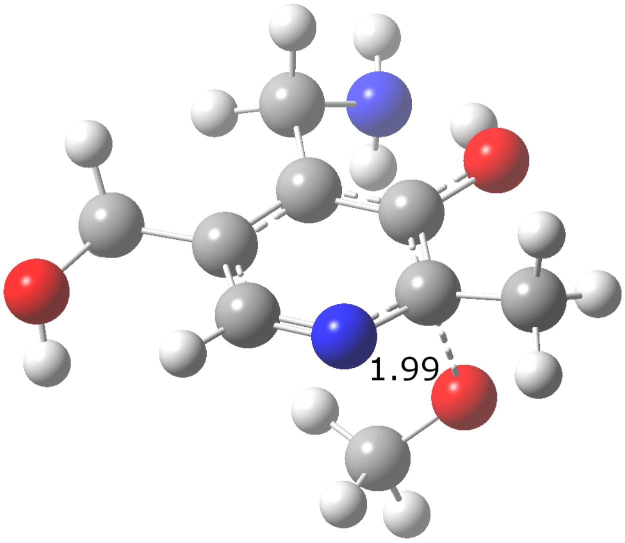

Charge=0, Multiplicity=2

C	-0.893618	0.596040	-0.466121
C	0.471316	0.827416	-0.274386
C	1.325396	-0.274839	-0.310522
C	0.774337	-1.538744	-0.584253
N	-0.511323	-1.767873	-0.770693
H	1.435339	-2.389481	-0.686525
C	0.978564	2.245090	-0.131466
H	1.972849	2.253647	0.314215
H	1.065036	2.684521	-1.127803
C	2.816894	-0.112116	-0.123845
H	3.231471	0.463237	-0.951970
H	3.012976	0.448482	0.794271
O	-1.781429	1.587003	-0.521625
H	-1.333985	2.383151	-0.105952
O	3.526836	-1.338430	-0.108945
H	3.290867	-1.821102	0.688176
C	-1.381985	-0.744226	-0.613530
N	0.023275	3.065957	0.630941
H	0.044534	2.822279	1.614155
H	0.251457	4.048275	0.552013
C	-2.780878	-0.957928	-1.099866
H	-3.055317	-1.995930	-0.927694
H	-2.835015	-0.749591	-2.171123
H	-3.471666	-0.300595	-0.580142
O	-1.864951	-0.798757	1.318336
C	-0.874916	-1.369583	2.103188
H	-1.254599	-1.377352	3.134134
H	-0.643656	-2.401997	1.821952
H	0.051724	-0.779329	2.113235

TS-HPM(0)(OCH_3,C3)●

Charge=0, Multiplicity=2

C	0.330599	-2.024625	-0.009296
C	1.141684	-0.889890	0.060362
C	-0.809471	0.382698	-0.541739
C	-1.532410	-0.856748	-0.633361
N	-0.969716	-2.010950	-0.341895
H	0.757260	-2.996781	0.205754
C	-2.976572	-0.805134	-0.993328
H	-3.111258	-0.413336	-2.003040
H	-3.403973	-1.802526	-0.928025
H	-3.496809	-0.125996	-0.314453
C	2.587766	-1.037070	0.435660
H	2.808518	-0.439270	1.324788
H	2.794810	-2.083329	0.667451
O	3.391088	-0.598088	-0.661631
H	4.313115	-0.642882	-0.395156
C	0.587686	0.342566	-0.244317
C	1.385133	1.620199	-0.240292
H	2.223355	1.523741	0.451548
H	1.809681	1.767092	-1.235407
O	-1.334878	0.835876	1.303144
O	-1.356142	1.442174	-1.146647
N	0.526005	2.772319	0.065438
H	0.159553	2.696522	1.007176
H	1.041382	3.637366	-0.022103
H	-0.812171	2.226371	-0.847725
C	-1.321022	-0.189019	2.244524
H	-2.109890	-0.932021	2.080587
H	-0.352956	-0.706894	2.284148
H	-1.475539	0.264795	3.231450

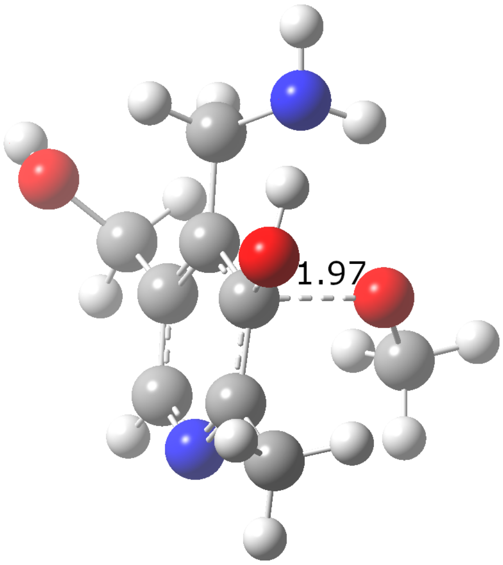

TS-HPM(0)(OCH_3,C4)•

Charge=0, Multiplicity=2

C	0.890416	-1.720577	-0.286735
C	1.186402	-0.387322	-0.387817
C	-1.201019	0.007870	-0.415147
C	-1.390977	-1.386377	-0.296976
N	-0.370312	-2.217680	-0.234332
H	1.684017	-2.454331	-0.262485
C	-2.786085	-1.930012	-0.259927
H	-3.348149	-1.496578	0.569029
H	-3.324161	-1.683228	-1.176567
H	-2.745497	-3.010490	-0.145852
C	2.606976	0.116135	-0.415815
H	2.822535	0.582593	-1.377872
H	2.723390	0.878939	0.358633
O	3.574039	-0.907427	-0.254250
H	3.503257	-1.251938	0.640375
C	0.111016	0.552676	-0.379983
O	-2.277196	0.784384	-0.512802
H	-1.935527	1.741793	-0.506794
C	0.336582	1.974618	-0.846199
H	0.339259	1.975051	-1.938227
H	1.310819	2.321479	-0.504274
N	-0.750888	2.852166	-0.389437
H	-0.641934	3.023138	0.602700
H	-0.730296	3.737080	-0.878625
O	0.189401	1.099143	1.540125
C	-0.216182	0.090408	2.414314
H	0.090765	0.404091	3.419583
H	-1.300467	-0.060447	2.417600
H	0.287659	-0.861871	2.210281

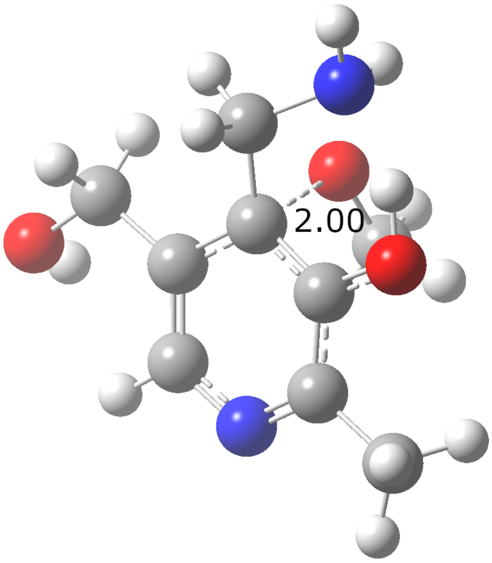

TS-HPM(0)(OCH_3,C6)•

Charge=0, Multiplicity=2

C	-1.180201	0.267748	0.756232
C	-0.403316	-0.860606	0.398512
C	1.386557	0.649283	-0.039109
C	0.548630	1.725729	0.363498
N	-0.679442	1.525437	0.782473
H	-2.089330	0.117002	1.319316
C	1.095038	3.118365	0.331143
H	1.990585	3.196890	0.949105
H	1.381194	3.396444	-0.684482
H	0.337578	3.807741	0.695616
C	-1.067310	-2.215665	0.336029
H	-0.854704	-2.662416	-0.641183
H	-0.646816	-2.877036	1.096299
O	-2.459961	-2.155439	0.553926
H	-2.783975	-1.462368	-0.046374
C	0.905619	-0.668160	-0.009840
C	1.772001	-1.812190	-0.487660
H	1.421753	-2.752831	-0.063657
H	1.681486	-1.886319	-1.573531
O	2.622736	0.949658	-0.444406
H	3.126723	0.077172	-0.465741
O	-2.348095	0.147306	-0.925280
C	-3.159894	1.285289	-0.915574
H	-3.753614	1.372816	0.001461
H	-2.587862	2.205198	-1.061565
H	-3.858105	1.182434	-1.755967
N	3.190316	-1.564057	-0.175193
H	3.372364	-1.726262	0.808389
H	3.786421	-2.189076	-0.701947

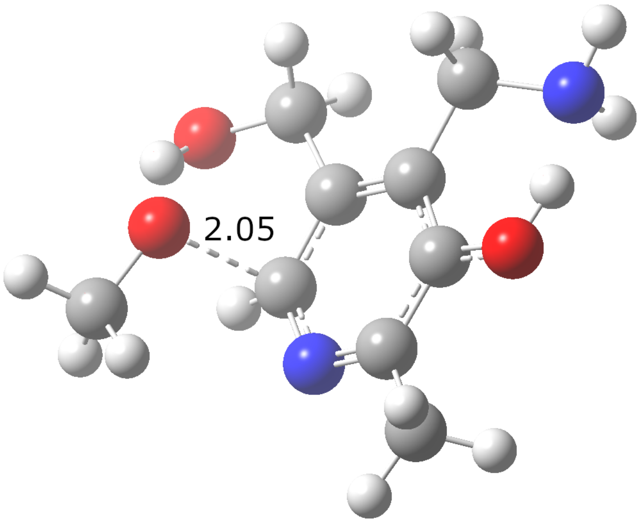

Supplement: Supplementary file 1 [file antioxidants-08-00344-s001.pdf]
